# Natural antioxidants that act against Alzheimer’s disease through modulation of the NRF2 pathway: a focus on their molecular mechanisms of action

**DOI:** 10.3389/fendo.2023.1217730

**Published:** 2023-07-03

**Authors:** Grammatiki Alexandra Sidiropoulou, Athanasios Metaxas, Malamati Kourti

**Affiliations:** ^1^ Angiogenesis and Cancer Drug Discovery Group, Basic and Translational Cancer Research Centre, European University Cyprus, Nicosia, Cyprus; ^2^ Department of Life Sciences, School of Sciences, European University Cyprus, Nicosia, Cyprus

**Keywords:** Alzheimer’s disease, antioxidants, NRF2, oxidative stress, reactive oxygen species, neurodegenerative diseases, neuroprotection

## Abstract

Characterized by a complex pathophysiology that includes the intraneuronal formation of neurofibrillary tangles and the extracellular deposition of β-amyloid plaques, Alzheimer’s disease (AD) is a terminal neurodegenerative disease that causes dementia in older adults. Oxidative stress in the brain is considered as one of the contributing factors to the pathogenesis of AD, and thus, antioxidants have attracted much interest as potential therapeutic agents against the disorder. Natural antioxidants are typically characterized by low acute and chronic toxicity, which facilitates their potential therapeutic application. One important molecular target for the beneficial effects of natural antioxidants is the nuclear factor erythroid-derived 2-related factor 2 (NFE2L2/NRF2). NRF2 is a key transcription factor that orchestrates the cellular antioxidant response through regulating the expression of oxidative stress-related genes harboring the antioxidant response element (ARE) in their promoters. Indeed, in the case of excessive oxidative damage, NRF2 migrates to the nucleus and binds to ARE, activating the transcription of antioxidant protector genes. There is increasing evidence that NRF2 is implicated in AD pathology through dysfunction and altered localization, which renders it as a potential therapeutic target for AD. Thus, this review summarizes the most recent (2018-2023) advances on the NRF2-modulating activity of natural antioxidants observed *in vitro* and in AD animal models. This information will help elucidate the molecular mechanisms governing the antioxidant activity of such phytochemicals to highlight their therapeutic potential against common neurodegenerative diseases, such as AD.

## 1 Introduction

Alzheimer’s disease (AD) is a progressive neurodegenerative disorder that is clinically characterized by cognitive and behavioral impairment, and is considered to be caused by a variety of genetic, epigenetic and environmental factors. The disease is named after Alois Alzheimer, a Bavarian psychiatrist specializing in neuropathology, whose work in the early 20^th^ century significantly altered the way mental disorders are perceived (1). AD commonly appears in older age (>65 years), and is one of the leading causes of dementia worldwide, accounting for up to 70-80% of all dementia cases (2). Although AD is currently viewed as a complex, multifactorial disorder, its exact pathophysiology and molecular mechanisms are still not thoroughly understood (3, 4).

Histopathologically, AD is characterized by the deposition of β-amyloid (Aβ) plaques extracellularly and the accumulation of neurofibrillary tangles (NFTs) intracellularly. The presence of these lesions in the cortex and hippocampus after examination of the brain in imaging studies has been associated with progressive amnesic dementia and impairment of several cognitive functions (2).

An important factor that aggravates the accumulation of Aβ and NFTs, and therefore contributes to the onset and progression of AD, is oxidative stress (5, 6). Oxidative stress is a pathological condition which is attributed to a disturbance in the balance of the endogenous and exogenous antioxidant mechanisms and is characterized by an excessive production of free radicals, including reactive oxygen species (ROS). ROS in high concentrations can interact with cellular DNA of the nucleus and mitochondria, proteins and lipids through various mechanisms and cause disruption of their homeostasis, ultimately leading to insufficient functioning of the antioxidant mechanism, *i.e.* oxidative stress (7). Numerous studies have supported and demonstrated that oxidative stress significantly contributes to AD progression (8–11). The central nervous system (CNS) consumes 20% of total oxygen and is particularly vulnerable to oxidative damage, especially in the presence of high levels of unsaturated fatty acids (12). This is due to the biology of neurons, which are non-dividing, post-mitotic cells that lack the ability to replicate and are not replaced upon damage, leading to accumulation of mitochondrial dysfunction over their lifespan (13). AD brains show an excess of ROS as well as bioactive metals such as copper, iron, zinc and magnesium, which are capable of further promoting oxidative damage and exacerbating the pathophysiology of the disease (14).

Nuclear factor erythroid 2-related factor 2 (NRF2) is a very important transcription factor that regulates the expression of a large number of genes related to oxidative stress, mitochondrial biogenesis, mitophagy and mitochondrial function (15). In the context of neurodegeneration, including AD, altered function and cellular mis-localization of NRF2 in the cytoplasm instead of the nucleus have been observed (16). The activation of NRF2 induces protective effects against oxidative damage through upregulation of antioxidant defenses, inhibition of inflammation and maintenance of protein homeostasis, and for this reason it has emerged as a new therapeutic target against AD (17). Indeed, recent studies and novel treatment strategies aimed at reducing oxidative stress through the use of phytochemicals that act as NRF2 activators have shown therapeutic effects in AD mouse models and in neuroblast-rich cell lines, such as PC12 (18, 19).

This literature review is designed to examine the latest data in relation to the role of oxidative stress in the pathogenesis and progression of AD, as well as the new innovative treatment strategies based on strengthening the body’s antioxidant defenses through activation of the NRF2 pathway by phytochemicals.

## 2 Alzheimer’s disease

### 2.1 Molecular characteristics and pathogenesis

**Figure 1:**
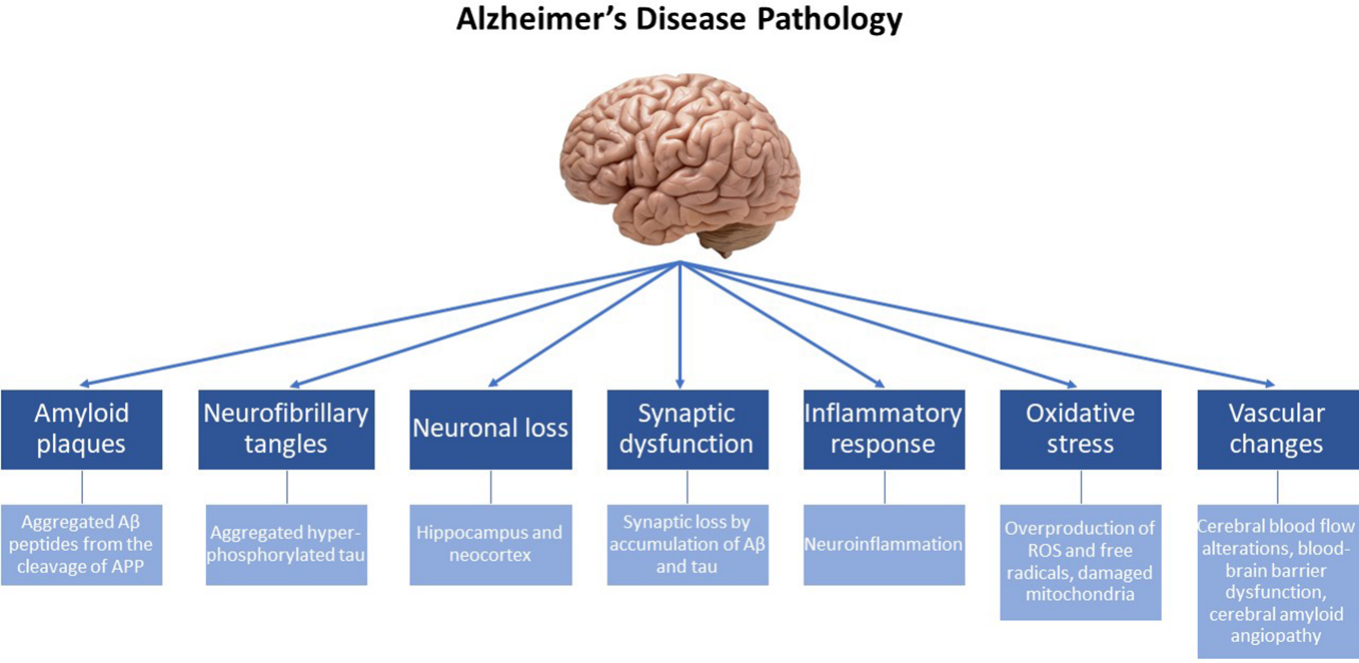
Alzheimer’s disease (AD) is a neurodegenerative disorder characterized by progressive cognitive decline and memory impairment. The underlying pathological changes in the brain of individuals with AD include the following key features: Amyloid plaques: Extracellular deposits of beta-amyloid protein (Aβ) form amyloid plaques. These plaques are composed of aggregated Aβ peptides derived from the cleavage of the amyloid precursor protein (APP). Neurofibrillary tangles: Intracellular neurofibrillary tangles are formed by the hyperphosphorylation and aggregation of tau protein. In healthy neurons, tau helps maintain the structural integrity of microtubules, but in AD, abnormal tau protein forms twisted filaments, leading to the formation of tangles. Neuronal loss: AD is associated with significant neuronal loss, particularly in brain regions involved in memory and cognitive functions, such as the hippocampus and neocortex. Neuronal loss contributes to the progressive decline in cognitive abilities. Synaptic dysfunction: Synaptic loss and dysfunction occur early. Disruptions in the communication between neurons impair memory formation and retrieval. Synaptic loss is believed to be caused by the accumulation of Aβ and tau pathology. Inflammatory response: AD is characterized by chronic neuroinflammation. Activated microglia and astrocytes are observed in affected brain regions, releasing pro-inflammatory molecules that contribute to neuronal damage. Oxidative stress: The increased production of free radicals and ROS, along with the overexpression of prooxidant enzymes and the dysfunction of mitochondria, contribute to chronic oxidative stress observed in the brain, that is intertwined with chronic neuroinflammation. Vascular changes: Vascular abnormalities are frequently observed in AD. Cerebral blood flow alterations, blood-brain barrier dysfunction, and the accumulation of vascular deposits, known as cerebral amyloid angiopathy, are seen in affected individuals.

Depending on the age at onset, AD can present either before (early-onset) or after the age of 65 (late-onset). Previous studies have demonstrated that the prevalence of early-onset AD is rather low (< 5%), and commonly associated with autosomal-dominant genetic mutations, which include mutations in the genes encoding for the *amyloid-beta precursor protein* (*APP*) (20, 21) as well as in the *presenilin 1* (*PSEN1*) and *2* (*PSEN2*) genes (22). Additionally, the etiology of early-onset AD has been attributed to the duplication of the *APP* locus as well as to trisomy of chromosome 21, which causes Down syndrome (23). The etiology of the most common, late-onset disease (sporadic AD) is basically attributed to a variety of genetic and environmental factors. Among them, the ϵ4 allele of the *Apolipoprotein E* (*APOE*) gene (24), has been documented to increase the probability of late-onset AD significantly more than various other predisposition genes studied and described in the literature (25, 26). Additional risk genes that contribute to late-onset AD have been recently identified by genome-wide association studies (GWAS), some of them being involved in processes of neuroinflammation (*TREM2*, *TYROB* and *CD33*), memory (*CR1*, *PICALM* and *BIN1*) and lipid metabolism (*ABCA7* and *CLU*) (4). From a molecular point of view, it is important to note that both early- and late-onset AD are essentially characterized by the same traits, *i.e.* the presence of plaques and tangles (the underlying pathological changes in the brains of AD patients are summarized in **Figure 1**).

#### 2.1.1 β-Amyloid and Alzheimer’s disease

Despite the fact that various factors have been implicated in the induction and pathophysiology of AD, the deposition of Aβ is considered to be the most important in promoting disease pathogenesis. The Aβ consists of polypeptides of various lengths. The two lengths most commonly found in plaques are composed of 40 or 42 amino acids. The Aβ_40_ form is abundant in plaques, but Aβ_42_ is the form that aggregates faster (27).

Aβ production is initiated by the sequential cleavage of APP by β-secretases (Beta-Secretase 1, BACE1) and γ-secretases, which eventually produce insoluble Aβ fibrils. Aβ has the ability to oligomerize, diffuse into synaptic clefts, and interfere with synaptic signaling (2). Aβ also polymerizes into insoluble fibrils, which aggregate into plaques. The polymerization of Aβ can lead to the activation of kinases responsible for tau protein hyperphosphorylation. Plaque aggregation is followed by recruitment of microglia around the plaques, which promotes microglial cell activation and local inflammatory responses, further contributing to neurotoxicity (3). According to recent studies (28, 29), it is argued that the toxicity caused to neurons is primarily due to soluble Aβ oligomers (soluble Aβ, sAβ) and to a smaller extent due to the mature fibrils. Cell-released sAβs cause neuronal dysfunction *in vivo* as well as synaptic loss, by modulating pathways which play a crucial role in neuronal survival and synaptic plasticity (30). The aforementioned sequence of events is known as the amyloid cascade hypothesis for the pathogenesis of AD, and is supported by studies that demonstrate the existence of sAβs well before Aβ deposition (31), as well as the detrimental effects of sAβs on hippocampal long-term potentiation, which is considered to be the cellular basis of learning and memory (32).

Aβ plaques initially develop in parts of the cerebral cortex, including the basal, temporal, and orbital regions of the frontal lobe, and at later stages progress throughout the neocortex, hippocampus, amygdala, diencephalon, and basal ganglia. In more critical cases, the plaques spread throughout the midbrain as well as the cerebellar cortex (3).

#### 2.1.2 Structure and function of APP

APP is a member of a family of related proteins that includes the mammalian amyloid precursor-like proteins 1 and 2 (APLP1, APLP2) and the amyloid precursor protein in Drosophila (3). It is a transmembrane protein with a long extracellular N-terminus and a shorter cytoplasmic C-terminus. As for the human *APP* gene, it is located on chromosome 21 and is expressed in all tissues, with the highest expression occurring in the CNS. APP plays an important role in various functions such as neuronal differentiation, neurogenesis, synaptic function, cell proliferation and apoptosis (33, 34). Alternative splicing promotes the generation of approximately ten variants of APP that encode APP isoforms of 639-770 amino acids. The three major isoforms are APP695, APP751 and APP770, and are capable of generating Aβ after sequential cleavage by β- and γ-secretases. Of the three, APP695 is the major isoform in the human brain, as it is mainly expressed in neurons and its expression increases significantly during neuronal differentiation, while the other two isoforms are mainly expressed in non-neuronal cells (35). APP overexpression appears to be involved in the pathogenesis of the disease as it results in an increase in Aβ levels. For example, studies have suggested that in rare cases of duplication of the APP locus or in cases of trisomy 21 (Down syndrome) individuals go on to develop early-onset AD (36) and show increased Aβ production in the brain (37).

APP cleavage and degradation are very important processes for disease progression. APP undergoes proteolytic cleavage through amyloidogenic and non-amyloidogenic processing. The majority of APP (90%) is cleaved through non-amyloidogenic processing, which includes activation of α-secretase, proteolytic cleavage within the APP Aβ peptide sequence, and generation of an N-terminus cleaved by α-secretase, sAPPa (soluble secreted amyloid precursor protein alpha). The α-secretase activity is attributed to the ADAM (a disintegrin and metalloproteinase) family of proteins, which provide neuroprotective effects by increasing neuronal plasticity and survival in cases of cytotoxicity and by proliferation of neural stem cells (38). sAPPa is thought to have neuroprotective activity, which is counteracted by Aβ oligomers, as discussed above, since they disrupt the PI3K (phosphoinositide-3 kinase)/Akt (protein kinase B) signaling pathway, leading to the inactivation of Akt and subsequent activation of glycogen synthase kinase-3β (GSK-3β). The dysregulation of this pathway ultimately results in impaired neuronal function and increased vulnerability to neurodegeneration (30).

Amyloidogenic processing of APP involves two sequential cleavages by β-secretases and γ-secretases. BACE1 cleaves APP, generating an extracellular soluble fragment (soluble secreted amyloid precursor protein beta, sAPPβ) and an intracellular C-fragment (CTF-99). sAPPβ is a peptide that acts as an agonist on death receptor 6 (DR6) and causes the activation of caspase 6, resulting in the stimulation of axonal pruning and consequently the death of neuronal cells (39). CTF-99 is further cleaved within the membrane by γ-secretase. This enzyme produces Aβ fragments of different lengths, mainly including Aβ_40_ and Aβ_42_. Peptides released by cleavage at the γ-site are then free to undergo oligomerization and subsequent fibrillation to form the characteristic plaques found in the atrophic brains of AD patients. Aβ_42_ is considered more toxic because it is more prone to oligomerization and this enhances its ability to exert neurotoxic actions (40). Additionally, according to recent research, the accumulation of CTF-99 stimulates an excessive alteration in the morphology of mitochondria leading to increased production of ROS, further contributing to their abnormal function and the progression of disease (41).

#### 2.1.3 Aβ accumulation and clearance

Aβ accumulation and clearance depend on the allele of *APOE* encoding apolipoprotein E, which plays a primary role in lipid transport. Currently, the exact mechanism of *APOE* in AD is not fully understood (42).

Four alleles of *APOE* have been identified. Of these, the *APOEϵ4* allele is present in 14.5% of the general population and 40% of patients with late-onset AD, while the *APOEϵ2* allele is present in 6.4% of the population. GWAS and meta-analyses have shown that the *APOEϵ4* allele is considered the strongest genetic risk factor associated with sporadic AD since its discovery in 1993, while the relatively rare allele *APOEϵ2* is considered the strongest genetic protective factor against AD (43, 44). The different effects of *APOE* isoforms on AD risk may be mediated by their differential impact on the deposition of Aβ plaques. The above neuropathological correlations support the suggestion that *APOE* promotes the accumulation and deposition of Aβ in insoluble fibrillar plaques, a suggestion that has been corroborated by several studies, including studies in mice with cerebral β-amyloidosis, in which a reduction in Aβ plaque burden has been observed when genetic deletion or deficiency of *APOE* is present (43). Furthermore, *in vivo* and *in vitro* studies have shown that *APOEϵ4* is involved in the inhibition of Aβ clearance from the brain, thereby prolonging its half-life in the interstitial fluid and thus, enhancing its toxicity (45).

#### 2.1.4 Tau protein and Alzheimer’s disease

Tau protein is very important for the function of neurons as it contributes to the polymerization and stability of axonal microtubules in the brain, facilitating the transport of organelles and enzymes along the cytoskeleton, thus contributing to the regulation of axonal growth and axonal transport. Tau protein undergoes numerous post-translational modifications, the most important being phosphorylation by kinases such as JNK (c-Jun N-terminal kinase), AMPK (5’ AMP-activated protein kinase), GSK-3β and others. Tau possesses a large number of potential phosphorylation sites due to the large number of residues that undergo serine and threonine phosphorylation, rendering it easily modifiable through this process (46). Tau protein phosphorylation controls both its normal and pathological functions. Normally, phosphorylation is regulated during development, in which fetal tau is more phosphorylated than adult brain tau. Hyperphosphorylation of the protein leads to a pathological state characterized by high aggregation, which then forms oligomers and ultimately leads to the formation of helical or straight phosphorylated tau neurofibrils. The resulting fibrils reduce the affinity of tau protein for microtubules, thereby further contributing to neurotoxicity (47). Tau protein-mediated toxicity occurs through two main mechanisms. One mechanism is based on the toxic loss of protein function, which results in the destabilization of microtubules, while the second is based on the toxic function of the protein, where the highly phosphorylated form of tau (p-tau) exerts toxic effects (48).

### 2.2 Oxidative stress and its role in the pathogenesis and progression of Alzheimer’s disease

Oxidative stress has been shown to be a dominant factor in the progression of AD. Over the years, significant oxidative damage accumulates in the neuronal tissue of AD patient brains, caused by the overproduction of free radicals (49). Free radicals, including ROS, are highly reactive molecules that have unpaired electron(s) in their outer shell, making them highly unstable. They are normally produced by metabolic processes of living organisms such as cellular oxidation, mitochondrial function, regulation and signaling, while they are also produced by specific cells of aerobic organisms such as macrophages, as well as by the electron transport chain in peroxisomes (50). The unpaired electron makes them particularly eager to attack other nearby molecules until a pair is formed. This results in free radical chain reactions and their propagation within the cell, jeopardizing vulnerable biomolecules, such as DNA, lipids and proteins.

The body’s antioxidant activity includes the endogenous and exogenous systems. The endogenous antioxidant system includes both enzymatic and non-enzymatic processes, which are regulated by endogenous molecules such as superoxide dismutase (SOD), catalase (CAT), glutathione peroxidase (GPx), and glutathione (GSH), which work by repairing damage resulting from free radicals or by scavenging free radicals. Exogenous antioxidants, such as vitamins C and E, carotenoids and polyphenols, are assimilated into the body through diet and are capable of repairing free radical damage extracellularly, as well as acting as cofactors of endogenous enzymatic antioxidants, enhancing repair or maintaining cellular redox homeostasis (51). Oxidative stress contributes to the pathogenesis and progression of AD in three main ways, as discussed below, which are macromolecule redox modifications, metal ion redox potential, and mitochondrial dysfunction, thus affecting cellular homeostasis, ROS generation, and upregulating the formation of Aβ and tau fibrils (6). Additionally, oxidative stress can further enhance the pathophysiological characteristics of AD by activating JNK and AMPK, which regulate tau phosphorylation and could exacerbate the accumulation of NFTs (52, 53).

#### 2.2.1 Redox regulated modifications of macromolecules

Macromolecule redox modifications initially involve the peroxidation of neuronal lipid double bonds, which results in the generation of biochemically active lipids that are highly reactive molecules, able to induce phosphorylation, tau protein dysfunction (54), disruption of the intracellular Ca^2+^ signaling pathway, and activation of apoptotic pathways (55). Nucleotides of nuclear and mitochondrial DNA are also particularly sensitive to oxidative damage, as they can undergo oxidative alterations by ROS such as hydroxylation, carbonylation and nitration (56). Finally, protein glycosylation and the production of advanced glycation end products (AGEs) have been shown to be accelerated in the presence of ROS (57). AGEs bind to the receptor for advanced glycation end products, RAGE, and induce the release of inflammatory mediators (58). Tau proteins can also be glycosylated and converted into AGEs, which leads to the inhibition of their ability to bind to microtubules and contributes to the formation of toxic NFTs (59). Similarly, in case of glycosylation of Aβ monomers, AGEs are again formed and the toxic accumulation and deposition of Aβ plaques are enhanced (42).

#### 2.2.2 Redox potential of metal ions

Copper, zinc and iron ions are very important for the biological functions of metalloproteins as well as for neuronal processes. In the case of neurodegenerative diseases, such as AD, there is a disruption of metal ion homeostasis in the brain (60). Zinc and copper cations bind to the hydrophilic N-termini of Aβ peptides and undergo continuous redox reactions, promoting the generation of ROS and causing a vicious cycle of increased oxidation and ROS production, which results in the accumulation of toxic Aβ peptides that cannot exit the synapse. Similarly, zinc ions interact with tau proteins promoting their phosphorylation through upregulation of tau-phosphorylating kinases, such as GSK-3β, and inhibition of phosphatases responsible for tau dephosphorylation (61). Finally, iron ions bind to tau protein monomers, enhancing the accumulation and formation of tau oligomers and subjecting them to continuous redox reactions, thereby increasing oxidative stress inside the cell (62, 63).

#### 2.2.3 Mitochondrial damage

Mitochondrial dysfunction is one of the initial cellular changes that occur during the progression of AD and is considered an important molecular feature of the disease (64). Healthy mitochondria are critical organelles that are responsible for ATP production, metabolism, stress responses and maintaining protein homeostasis. In AD, the accumulation of high levels of Aβ and p-tau and the overexpression of tau are directly related to the generation of mitochondrial damage, as demonstrated by several recent studies (65–67). One of the important consequences of these processes is that they induce ROS generation causing excessive mitochondrial fragmentation and promoting defective mitophagy (68, 69). Mitophagy is the selective degradation of mitochondria through autophagy, which aims to maintain the homeostasis of the cell and prevent the accumulation of dysfunctional mitochondria that can lead to cellular degeneration. It is regulated by several proteins including Parkin and PTEN-induced kinase 1 (PINK1), whose mutations have been observed in patients with neurodegenerative diseases such as Parkinson’s disease and AD (70). In the brains of AD patients, the main mitochondrial lesions that occur and lead to defective mitophagy are: age-dependent accumulation of mitochondrial DNA (mtDNA) mutations, reduced mitochondrial membrane potential, increased mitochondrial ROS production, reduced axonal transport, reduced ATP production, altered function of enzymes and increased fragmentation (71–73).

### 2.3 Altered NRF2 in Alzheimer’s disease

**Figure 2:**
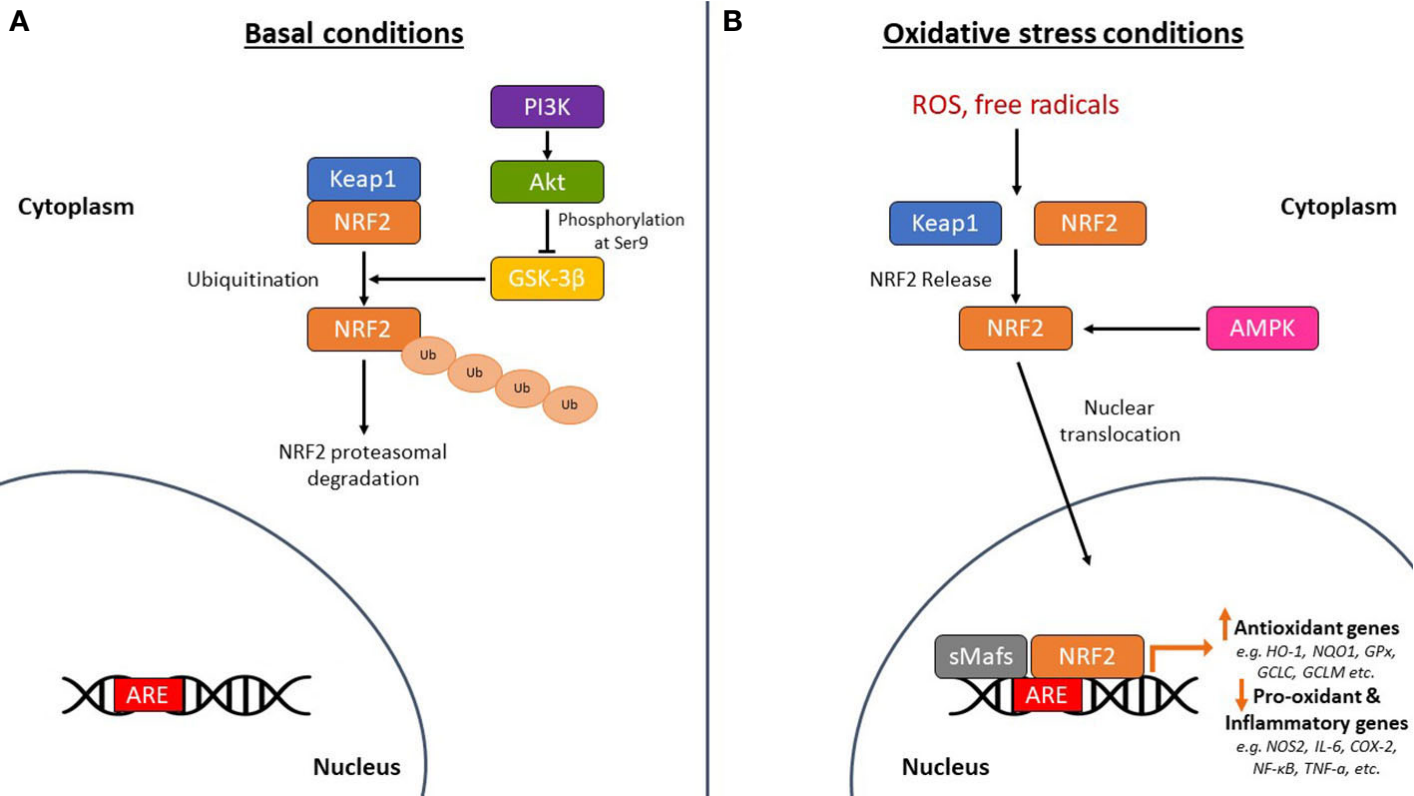
The mechanism of action of nuclear factor erythroid-derived 2-related factor 2 (NRF2) in basal and oxidative stress conditions. **(A)** Under basal conditions, NRF2 is sequestered by Kelch-like ECH-associated protein 1 (Keap1) in the cytoplasm leading to its ubiquitination and subsequent proteasomal degradation. Glycogen synthase kinase-3β (GSK-3β) phosphorylates NRF2 leading again to the proteasomal degradation of NRF2. The PI3K (phosphoinositide-3 kinase)/Akt (protein kinase B) signaling pathway phosphorylates GSK-3β at serine 9 to keep it in an inactive state, thereby protecting NRF2 from proteasomal degradation. **(B)** Under oxidative stress conditions, NRF2 is released from Keap1 and is translocated to the nucleus where it dimerizes with small musculoaponeurotic fibrosarcoma proteins (sMafs) and binds to specific DNA regions, the antioxidant response element (ARE) sequences. This regulates the expression of antioxidant genes, such as heme oxygenase-1 (HO-1), NAD(P)H quinone oxidoreductase 1 (NQO1), glutathione peroxidase (GPx), glutamate-cysteine ligase catalytic subunit (GCLC), and glutamate-cysteine ligase regulatory subunit (GCLM). It also downregulates the expression of pro-oxidant and pro-inflammatory genes, such as nitric oxide synthase 2 (NOS2), interleukin (IL)-6, cyclooxygenase 2 (COX-2), nuclear factor kappa beta (NF-κB), and tumor necrosis factor-a (TNF-a). 5’-Adenosine monophosphate-activated protein kinase (AMPK) is also linked to the phosphorylation and direct activation of NRF2 nuclear translocation, tuning the transactivation of antioxidant genes. *ROS, reactive oxygen species; Ub, ubiquitin.*

**Figure 3:**
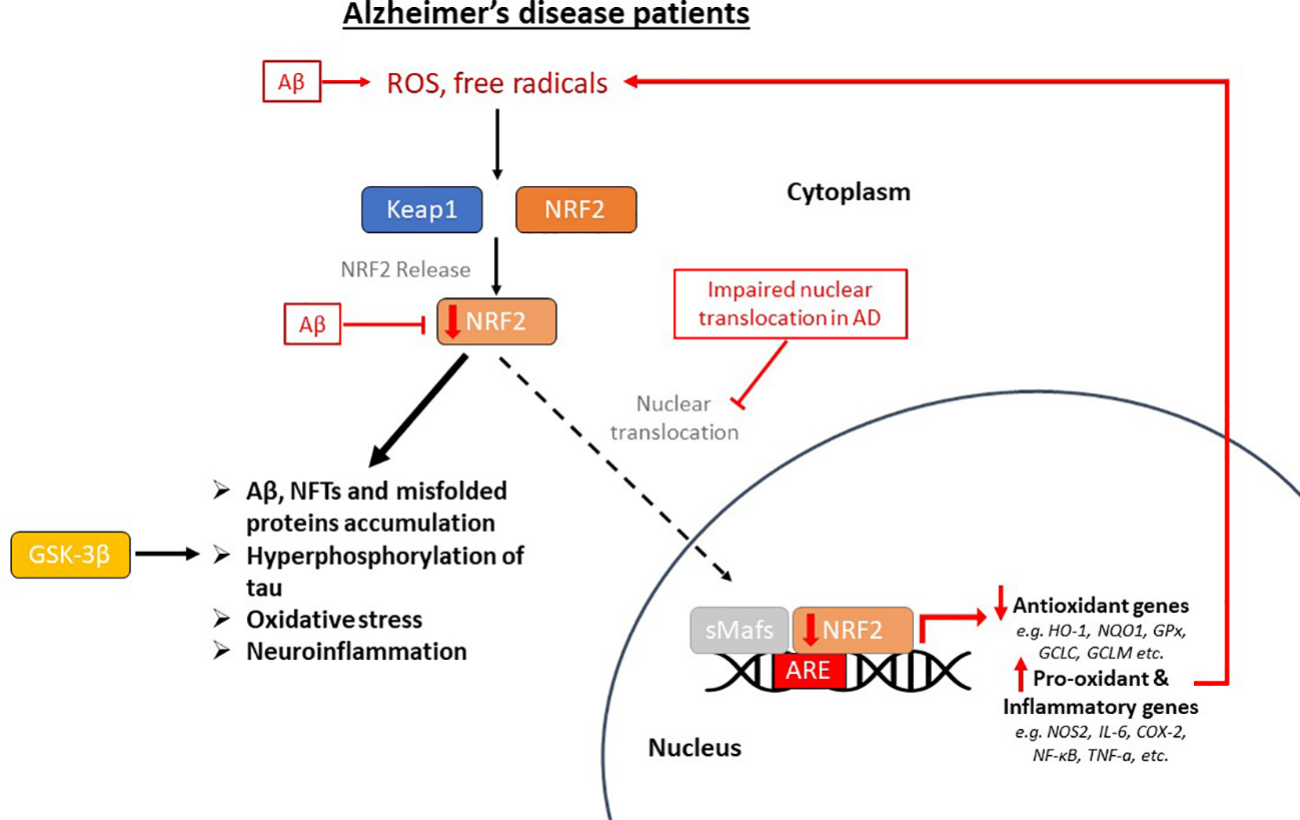
The impaired pathway of nuclear factor erythroid-derived 2-related factor 2 (NRF2) in Alzheimer’s disease (AD) patients. This pathway is dysregulated in AD, where oxidative stress in the brain positively regulates NRF2, but its nuclear translocation is inhibited, therefore there is decreased expression of antioxidant genes and overexpression of pro-oxidant and pro-inflammatory genes. Beta-amyloid (Aβ) plaques enhance oxidative stress, reduce NRF2 and inhibit its dissociation from Keap1 (Kelch-like ECH-associated protein 1). This altered regulation of NRF2 in the brains of AD patients leads to the accumulation of misfolded proteins, including the accumulation of Aβ and neurofibrillary tangles (NFTs), the hyperphosphorylation of tau, which is also carried out by GSK-3β (glycogen synthase kinase-3β), and its subsequent aggregation, further exacerbating oxidative stress and neuroinflammation. *ROS, reactive oxygen species; sMafs, small musculoaponeurotic fibrosarcoma proteins; ARE, antioxidant response element; HO-1, heme oxygenase-1; NQO1, NAD(P)H quinone oxidoreductase 1; GPx, glutathione peroxidase; GCLC, glutamate-cysteine ligase catalytic subunit; GCLM, glutamate-cysteine ligase regulatory subunit; NOS2, nitric oxide synthase 2; IL-6, interleukin-6; COX-2, cyclooxygenase 2; NF-κB, nuclear factor kappa beta; TNF-a, tumor necrosis factor-a.*

NRF2 is abundant in the human brain, primarily in microglia and astrocytes and secondarily in neurons (74). As an antioxidant transcription factor, NRF2 in astrocytes is inactive at basal conditions (**Figure 2A**), but enhances the expression of genes participating in GSH synthesis (75, 76) and downregulates pro-oxidant and pro-inflammatory proteins, including nitric oxide synthase 2 (NOS2), interleukin (IL)-6, cyclooxygenase 2 (COX-2) and tumor necrosis factor-a (TNF-a) (77) with the occurrence of oxidative conditions (**Figure 2B**). It has been shown that the levels and NRF2 activity are affected in the brains of people with neurodegenerative diseases, such as AD. In specific, NRF2 was found in the cytoplasm in the hippocampus of AD patients and its constitutive levels were significantly reduced in the nucleus (78). Similarly, a decrease in the mRNA levels of NRF2 was shown in the cortex of wild-type mice that was age-dependent (79). This finding was corroborated also in other studies, for example in the work of Kanninen et al. who observed that the amounts of Aβ dramatically increased while the levels of NRF2 significantly reduced as the mice aged. This was accompanied by decreased expression of three known targets of the NRF2 pathway, NAD(P)H quinone oxidoreductase 1 (NQO1), glutamate-cysteine ligase catalytic subunit (GCLC), and glutamate-cysteine ligase regulatory subunit (GCLM) (80). Bahn et al. demonstrated that, independent of oxidative stress, lowering NRF2 levels enhances BACE1 activity and, consequently, Aβ synthesis (81). This altered regulation of NRF2 in the brains of AD patients leads to the accumulation of misfolded proteins, including the accumulation of Aβ, the hyperphosphorylation of tau and its subsequent aggregation, further exacerbating oxidative stress (80, 82–84) (**Figure 3**).

### 2.4 Scope of this literature review

As previously discussed, there is evidence to suggest that NRF2 activity is altered in AD and its dysregulation may contribute to disease progression. NRF2 is a transcription factor that regulates the expression of genes involved in antioxidant cellular defense and inflammatory conditions (85). Several studies have reported that NRF2 activity is reduced in the brains of individuals with AD. The reduction in NRF2 activity is associated with increased levels of oxidative stress and inflammation in the brain. Specifically, it has been found that there is a decrease in the expression of genes that are regulated by NRF2, including genes involved in antioxidant defense and detoxification. Moreover, it has been suggested that the dysregulation of NRF2 activity may contribute to the accumulation of Aβ plaques and NFTs (16). For example, it has been shown that Aβ can inhibit NRF2 activity, which can lead to increased oxidative stress and inflammation (86). Overall, these findings suggest that NRF2 dysregulation may contribute to the pathogenesis of AD, and targeting NRF2 may have therapeutic potential for the treatment of this disease.

Therefore, conducting the present literature review was considered necessary as the treatment of AD is an unmet medical need which affects a large percentage of the aging population, while its pathophysiology is still not fully understood. For this reason, research to find innovative treatments based on newer findings is very important to achieve better outcomes for patients. The role of oxidative stress is considered key in AD progression as oxidative stress contributes significantly to aging and neurodegeneration, which highlights it as a good therapeutic target. The NRF2 transcription factor signaling pathway is an important pathway that activates antioxidant mechanisms in cells, which is impaired in patients with AD and its reactivation is considered to be of critical importance in the treatment of the disease. With this in mind, this literature review focuses on the most recent advances of the last 5 years in antioxidant phytochemicals that are capable of acting as NRF2 activators or potentiators with the aim of preventing or treating AD symptoms.

## 3 Phytochemical antioxidant compounds that act as activators of the transcription factor NRF2

### 3.1 Polyphenols

Polyphenols are a large group of naturally occurring compounds found in plants. They are characterized by the presence of multiple phenol groups in their chemical structure and can be further classified into various subclasses, including flavonoids, phenolic acids, stilbenes, and lignans (87). Polyphenols possess antioxidant and anti-inflammatory properties, and their actions are attributed to their ability to scavenge ROS, modulate enzymatic activity, and regulate gene expression. One of the mechanisms through which polyphenols exert their cellular effects is by activating NRF2 through various pathways, including: 1. Modification of Keap1 (Kelch-like ECH-associated protein 1), leading to the release and nuclear translocation of NRF2, where it binds to the antioxidant response element (ARE) in the DNA, thereby activating the expression of genes involved in antioxidant defenses (88). 2. Kinase signaling pathways: Polyphenols can activate kinases such as protein kinase C (PKC), mitogen-activated protein kinases (MAPKs), and PI3K/Akt, which in turn phosphorylate NRF2 or its negative regulator, resulting in the stabilization and nuclear translocation of NRF2 (89). 3. Epigenetic modifications: Some polyphenols can influence the activity of enzymes involved in DNA methylation and histone modification, leading to epigenetic changes that may affect NRF2 gene expression (90). Thus, polyphenols are potential NRF2 activators that could benefit the pathophysiology of AD.

Rosmarinic acid (RosA), a polyphenolic compound found in various culinary herbs, was used against Aβ-induced toxicity in PC12 cells, and the results showed that RosA (10 μM) attenuated Aβ-induced ROS production and reduced lipid hydroperoxides, activating the NRF2-induced cellular antioxidant defense system. More specifically, RosA enhanced the nuclear translocation of NRF2 and its nuclear binding to ARE, activating a set of NRF2 target genes encoding phase II antioxidant enzymes. Mechanistically, the antioxidant effects of RosA were mainly mediated by the Akt/GSK-3β/Fyn pathway through increased NRF2 activity (91).

A polyphenolic extract of *Arabidopsis thaliana* was tested *in vitro* on mouse BV-2 microglial cells treated with Aβ_25-35_ at a dose of 20 μl/ml. The extract caused a decrease in the production of pro-inflammatory cytokines (IL-6, IL-1β, TNF-a) and an increase in the production of anti-inflammatory cytokines (IL-4, IL-10, IL-13), while it also enhanced the activation of NRF2, leading to upregulation of heme oxygenase-1 (HO-1). The efficacy of the extract was also confirmed in a *Drosophila melanogaster* model expressing human Aβ_1-42_, where it ameliorated Aβ-induced motor dysfunction in flies, indicating neuroprotection *in vivo* (92).

*In vivo* studies in an AD experimental model of *Caenorhabditis elegans* (*C. elegans*) showed that treatment with a methanolic extract of Romina variety strawberry (100, 500, 1000 μg/mL) (93) and treatment with an olive extract with 20% hydroxytyrosol content (100 μg/mL) (94), both extracts rich in phenolic compounds, led to reduction of oxidative stress and Aβ accumulation, while delaying the Aβ-induced paralysis of *C. elegans*, without apparent toxicity at these doses. The RNAi technology applied in both studies showed that the efficacy of the methanolic extract of strawberry derives partially from the activation of the DAF-16/FOXO (Forkhead box protein O) and SKN-1 (protein skinhead-1)/NRF2 pathways, while the efficacy of the olive extract derives from the activation of the SKN-1/NRF2 and the overexpression of the *C. elegans* lifespan biomarker HSP-16.2 (heat shock protein 16.2).

According to various studies, caffeic acid (CA), along with its ester derivatives, has shown significant therapeutic effects against AD. In an *in vivo* and *in vitro* study by Colonnello et al. (95), rats and *C. elegans* were exposed to various toxins, and then administered CA at doses of 100 μM and 25 mM, respectively. The results showed that CA prevented cell dysfunction, oxidative damage and transcriptional regulation in the rat cortex, and restored the activity of NRF2/ARE, while in *C. elegans* it reduced the loss of survival and motor alterations caused by the toxins. The compounds CA phenethyl ester (CAPE) and CA phenethyl ester 4-O-glucoside (FA-97) were also examined for their potential activity against AD. CAPE showed a protective effect against the memory impairment induced by intracerebral injection of Aβ_O_ (amyloid-β oligomers) in mice, preventing the increase of ROS levels, neurodegeneration and neuroinflammation. Its administration was carried out at a dose of 10 mg/kg intraperitoneally (i.p.) after intracerebroventricular (i.c.v.) injection of Aβ_O_ and led to the neutralization of oxidative stress through the induction of NRF2/HO-1 pathway in the hippocampus of mice (96). FA-97 protected against hydrogen peroxide (H_2_O_2_)-induced apoptosis, decreased ROS and MDA (malondialdehyde) levels, and increased GSH and SOD levels in SH-SY5Y and PC12 cells through activation of NRF2/HO-1 signaling. *In vivo*, FA-97 prevented scopolamine-induced cognitive impairment by reducing neuronal apoptosis, protecting against cholinergic system dysfunction, and reversing elevated MDA level and low total antioxidant capacity (T-AOC) in mouse brains (97) (**Table 1**).

**Table 1:** Polyphenolic phytochemicals exerting neuroprotective activities in Alzheimer’s disease models.

Phytochemical tested	Plant	Concentration/Dose tested	Disease model	NRF2-related mechanism	Other observed AD-related effects	Reference
Rosmarinic acid	*Salvia Rosmarinus L.*	• 10 μM	• Aβ-induced PC12 cells	• Upregulation of NRF2 expression and nuclear translocation	• Decrease of ROS production and LPO • Increase of phase-II antioxidants	(91)
Polyohenolic extract of *Arabidopsis thaliana*	*Arabidopsis thaliana*	• 20 μl/ml	• Aβ_25-35_-induced BV-2 murine microglia cells • *Drosophila melanogaster* expressing human Aβ_1-42_	• Upregulation of NRF2 and HO-1 levels	• Increase of NQO1, IL-4, IL-10 and IL-13 • Decrease of IL-6, IL-1β and TNF-a	(92)
Strawberry methanolic extract	*Fragaria x ananassa* cv. *Romina*	• 100, 500 or 1000 μg/ml	• *C. elegans*	• Partial activation and involvement of SKN-1/NRF2 pathway	• Partial activation and involvement of DAF-16/FOXO pathway • Decrease of OS and Aβ aggregation	(93)
Olive oil extract 20% rich in Hydroxytyrosol	*Olea europea*	• 100 μg/ml	• *C. elegans*	• Upregulation of SKN-1/NRF2	• Decrease of OS and Aβ-induced paralysis • Decrease of Aβ and tau aggregation	(94)
Caffeic acid	Honeybee propolis	• 100 μM • 25 mM	• Rat cortical slices • *C. elegans*	• Restoration of NRF2/ARE levels	• Neuroprotection against 6-OHDA-induced toxicity	(95)
Caffeic acid phenethyl ester	Honeybee propolis	• 10 mg/kg (i.p)	• Aβ_O_-induced AD mouse model	• Upregulation of NRF2/HO-1 pathway	• GSK-3β modulation in hippocampus	(96)
FA-97, synthetic caffeic acid phenethyl ester derivative	Honeybee propolis	• 10 mg/kg • 0, 0.125, 0.25, 0.5, 1, 2 and 3 μM	• SCOP-induced AD mice • SH-SY5Y cells	• Promotion of NRF2 activity and nuclear translocation	• Upregulation of HO-1, NQO1, SOD and GSH • Decrease of MDA and ROS levels	(97)

Aβ, β-amyloid; AD, Alzheimer’s disease; NRF2, nuclear factor erythroid 2-related factor 2; ROS, reactive oxygen species; LPO, lipid peroxidation; HO-1, heme oxygenase-1; NQO1, NAD(P)H quinone oxidoreductase 1; IL-4, interleukin-4; IL-10, interleukin-10; IL-13, interleukin-13; IL-6, interleukin-6; IL-1β, interleukin- 1β; TNF-a, tumor necrosis factor a; C. elegans, Caenorhabditis elegans; SKN-1, protein skinhead-1, FOXO, forkhead box protein O; OS, oxidative stress; ARE, antioxidant response element; Aβ_O_, amyloid beta oligomers; GSK-3β, glycogen synthase kinase 3 beta; SCOP, scopolamine; SOD, superoxide dismutase; GSH, glutathione; MDA, malondialdehyde.

### 3.2 Flavonoids

Flavonoids are a diverse group of antioxidant polyphenolic compounds that are widely distributed in plants. They are characterized by a common structure consisting of two aromatic rings (A and B rings) connected by a three-carbon bridge (C ring). The C ring often contains oxygen atoms, forming various subgroups of flavonoids, including flavones, flavonols, flavanones, flavanols (catechins), and anthocyanins. Biologically, flavonoids exhibit anti-inflammatory, antioxidant, anti-cancer, cardioprotective, and neuroprotective activities (98). Flavonoids can modulate various cellular signaling pathways, gene expression, enzyme activities, and cellular functions, contributing to their biological effects, while they activate NRF2 signaling through similar mechanisms as polyphenols.

Quercetin is one of the main flavonoid compounds with potential therapeutic activity against various diseases, including neurodegenerative diseases (99). When administered i.p. at a dose of 50 mg/kg to a rat model of streptozotocin (STZ)-induced AD, it provided neuroprotection by activating the α7 nAChR (nicotinic acetylcholine receptor)/NRF2/HO-1 pathway, improving cognitive deficits, and reducing acetylcholinesterase (AChE) activity, Aβ aggregation and mitochondrial toxicity (100). A different study in a PC12 cell model that had undergone Aβ_25-35_-induced cytotoxicity, showed that the use of quercetin at different concentrations (0, 10, 20, 40 and 80 μmol/L) increased cell survival and proliferation, T-AOC, as well as the levels of the antioxidant enzymes SOD, GPx, CAT, sirtuin 1 and NRF2. It also reduced LDH (lactate dehydrogenase), AChE, MDA and HO-1 levels (101). In addition, the combination of quercetin with sitagliptin (Sita), using quercetin as pretreatment dissolved in olive oil at a dose of 100 mg/kg, showed good results in rats with Aβ-induced AD. The combination significantly increased escape latency in the Morris water maze (MWM) test of cognitive function, decreased Aβ_1-42_ levels, and increased NRF2/HO-1 expression and activity in rat brain (102).

Cyanidin-3-glucoside (C3G), a major anthocyanin found in berries, reduced L-glutamic acid (L-Glu)-induced oxidative damage and apoptosis, in mouse hippocampal HT22 neuronal cells, according to a study by Sukprasansap et al. (103). C3G exerted neuroprotective and antioxidant activities by inhibiting the expression of oxidative proteins, such as calpain and caspase-12, regulating the expression of survival proteins, such as NRF2 and ERK (extracellular signal-related kinase), and enhancing the activation of the endogenous antioxidant system, including proteins SOD, CAT, and GPx.

In another study using the APP/PS1 AD mouse model and HT22 cells, anthocyanin from Korean black beans supplementation was observed to control the PI3K/Akt/GSK-3 pathway which subsequently reduced Aβ_O_-induced oxidative stress and neurotoxicity by activating the NRF2 antioxidant system. The supplement improved memory capabilities and pre- and postsynaptic protein markers *in vivo*, highlighting it as a potent neuroprotective and antioxidant agent in AD (18).

Another flavonoid, folecitin, which is extracted from *Hypericum oblongifolium Wall*, was investigated for its therapeutic potential against lipopolysaccharide (LPS)-induced memory impairment caused by Aβ production in a mouse model. LPS was administered i.p. at a dose of 250 μg/kg/day for 3 consecutive weeks, followed by co-administration of folecitin (30 mg/kg/day) with LPS for the last two weeks. The results showed that folecitin significantly reduced LPS-induced apoptotic proteins, inhibited BACE1, and restored both presynaptic and postsynaptic connections, ameliorating memory impairment. Folecitin also activated the endogenous antioxidant proteins NRF2 and HO-1, by inducing phosphorylation of Akt pathway proteins (104).

Treatment with 10 µM and 20 µM isoliquiritigenin (ISL), a natural flavonoid found in licorice root, counteracted oxidative stress and inflammation in BV-2 cells stimulated with Aβ_O_, as it significantly reduced the production of inflammatory cytokines and nitric oxide (NO) and restored the morphological changes induced by Aβ_O_. In addition, it activated NRF2 signaling and suppressed nuclear factor kappa beta (NF-κB) signaling (105).

Nobiletin, a bioflavonoid derived from citrus peels, and hawthorn leaf flavonoids (HLF) were explored in different studies for their effect on Aβ- and Aβ_25-35_-induced toxicity, respectively, in rat models, where they showed equally significant therapeutic effects. Nobiletin was administered at a dose of 10 mg/kg/day from day 0 to day 7 after Aβ injection, and was shown to improve cognitive function and neuronal loss in the hippocampus, while reducing oxidative damage and neuroinflammation. These effects, according to the researchers, were partially attributed to the regulation of the toll-like receptor 4 (TLR4)/NF-κB/NRF2 pathways (106). HLF was administered at doses of 50, 100, and 200 mg/kg for a period of 30 days and showed a significant improvement in neuronal damage and memory deficits, an increase in the activity and levels of the antioxidant enzymes SOD, CAT, and GSH, and a decrease in MDA. HLF reduced oxidative stress specifically through activation of the NRF2/ARE signaling pathway (107).

Farrerol, an important bioactive component of rhododendron belonging to the class of flavones, was also found to be able to reduce Aβ-induced oxidative stress and inflammation in mouse BV-2 microglial cells at various concentrations (1.5 and 10 µM), through enhancing the activation of the NRF2/Keap1 pathway (108). Similarly, genistein, an isoflavone, was able to exert *in vitro* neuroprotective activity *via* the NRF2/HO-1/PI3K signaling in the SH-SY5Y cell line, which had undergone Aβ_25-35_-induced toxicity at doses of 10, 30, and 50 µM (109).

The methoxy-flavonoid, umuhengerin, was administered to an AD mouse model induced by intracerebral injection of STZ. Mice were orally administered umuhengerin at a dose of 30 mg/kg or donepezil as a positive control at a dose of 2.5 mg/kg for 21 days, and the results showed that umuhengerin attenuated STZ-induced neuroinflammation and oxidative stress by upregulating NRF2 and downregulating Keap1 expression, resulting in improved cognitive function. In addition, umuhengerin reduced the expression of β-secretase and NF-κB, as well as AChE activity, leading to reduced Aβ formation (110).

Hesperetin, a bioactive flavonoid produced from hesperidin, significantly reduced oxidative stress, apoptosis, and neurodegeneration *in vivo* at a dose of 50 mg/kg in the brain of mice that had received an i.c.v. injection of Aβ 24 h earlier. Similar results were also found *in vitro* at concentrations of 10, 20 or 50 µM hesperetin in HT22 and BV-2 cells challenged with Aβ, through reduction of ROS levels and lipid peroxidation and increase of NRF2/HO-1 expression (111).

Isoastilbin (IAB) was used against AD in a mouse model induced by AlCl_3_ and D-lactose, as well as in PC12 cells induced by L-Glu, and the results showed that it reduced oxidative damage in both models, while also reducing the deposition of Aβ and phosphorylated tau in the brain of mice. IAB further enhanced the nuclear levels of NRF2 and increased the expression of antioxidant enzymes regulated by it (112).

Finally, the flavonoid compound complanatoside A (CA), in an *in vivo* study, had the ability to extend the lifespan of *C. elegans* in a dose-dependent manner, with the 50 μM concentration exerting the maximum increase in lifespan by 16.87%. The mechanism found after further investigation was based on the involvement of DAF-16/FOXO, SKN-1 and heat shock factor 1 (HSF-1) signaling pathways. CA also caused a reduction in the aggregation of toxic proteins Aβ and α-synuclein, and improved the motility of the organisms (113) (**Table 2**).

**Table 2:** Flavonoid phytochemicals exerting neuroprotective activities in Alzheimer’s disease models.

Phytochemical tested	Plant	Concentration/Dose tested	Disease model	NRF2-related mechanism	Other observed AD-related effects	Reference
Quercetin	Fruits, vegetables, grains	• 50 mg/kg (i.p.)	• STZ-induced AD rats	• Upregulation of a_7_ nAChR/NRF2/HO-1 pathway	• Attenuation of STZ-induced cholinergic dysfunction in hippocampus, prefrontal cortex and amygdala of rats	(100)
Quercetin	Fruits, vegetables, grains	• 0, 10, 20, 40 and 80 μmol/L	• Aβ_25-35_-induced PC12 cells	• Increase of NRF2	• Increase of HO-1, SOD, GPx, CAT, T-AOC and sirtuin1	(101)
Quercetin + sitagliptin	Fruits, vegetables, grains	• 100 mg/kg p.o. dissolved in olive oil	• Aβ-induced Male Sprague–Dawley rats	• Upregulation of NRF2/HO-1 pathway	• Upregulation of SOD, CAT, GSH • Decrease of MDA and Aβ aggregation	(102)
Cyanidin-3-glucoside	Dark-colored fruits, plants and vegetables	• 0-100 μM	• Glutamate-induced HT22 mouse hippocampal neuronal cells	• Upregulation of NRF2 and ERK	• Suppression of calpain, caspase-12 and CHOP • Upregulation of SOD, CAT, GPx and phase II enzymes	(103)
Anthocyanin supplement	Korean black beans	• 25, 50, 100 μg/ml • 12 mg/kg/day i.p. for 30 days	• HT22 cells • APP/PS1 AD mouse model	• Activation of NRF2/HO-1 pathway	• Activation of PI3K/Akt/GSK-3β pathway • Reduced Aβ_O_-induced oxidative stress and neurotoxicity • Improvement of memory • Increased pre- and postsynaptic protein markers and decreased apoptotic markers; activated caspase-3 and PARP-1	(18)
Folecitin	*Hypericum oblongifolium*	• 30 mg/kg/day	• BALB/c mouse model induced by LPS	• NRF2 and HO-1 activation by stimulating Akt phosphorylation	• Decrease of LPS-induced apoptotic proteins BAX, PARP-1 and caspase-3 • Inhibition of BACE1	(104)
Isoliquiritigenin	*Licorice*	• 10 and 20 μM	• BV-2 cells stimulated with Aβ_O_	• NRF2 activation	• NF-kB suppression	(105)
Nobiletin	Citrus peels	• 10 mg/kg/day (p.o)	• AB-induced Wistar rats	• Upregulation of NRF2	• Decrease of MDA and ROS levels • Partial reversal of SOD activity • Downregulation of TLR4, NF-kB and TNF-a	(106)
Hawthorn Leaf flavonoids	*Crataegus pinnatifida*	• 50, 100 and 200 mg/kg p.o.	• Aβ_25-35_-induced AD mouse model	• Upregulation of NRF2/ARE	• Upregulation of HO-1, NQO1, SOD and CAT • Decrease of ROS and MDA	(107)
Farrerol	*Rhododendron dauricum L.*	• 1, 5, and 10 μM	• BV-2 microglial cells	• Enhancement of NRF2/Keap1 activation	• Decrease of ROS, MDA, IL-6, IL-1β and TNF-a • Attenuation of SOD inhibition	(108)
Genistein	*Genista tinctoria*	• 10, 30 or 50 μM	• Aβ_25-35_- induced SH-SY5Y cells	• Upregulation of NRF2/HO-1/PI3K pathway		(109)
Umuhengerin	*Lantana trifolia*	• 30 mg/kg p.o.	• STZ-induced AD mouse model	• Increase of NRF2 expression • Downregulation of Keap1	• Upregulation of HO-1 and GSH • Downregulation of NF-kB, p65, MDA, H_2_O_2_, AChE, TNF-a and BACE1	(110)
Hesperetin	Citrus fruits	• 50 mg/kg • 10, 20 or 50 μM	• Aβ mouse model • BV-2 cells • HT22 cells	• Upregulation of NRF2 both *in vivo* and *in vitro*	• Decrease of ROS and LPO • Regulation of TLR4 and p-NF-kB	(111)
Isoastilbin	*Hypericum perforatum*	• 10 or 30 μM • 40 mg/kg/day	• L-Glu-induced PC12 cells • AlCl_3_/D-galactose AD mouse model	• Upregulation of NRF2 nuclear levels	• Upregulation of SOD-1, CAT and HO-1 • Decrease of AChE levels, Aβ and tau expression and ROS levels	(112)
Complanatoside A	*Semen Astragalus Complanatus*	• 50 μM	• *C. elegans*	• SKN-1/NRF2 activation	• DAF-16/FOXO activation • Decrease of Aβ accumulation	(113)

i.p., intraperitoneally; STZ, streptozotocin; AD, Alzheimer’s disease; a7 nAChR, a7 nicotinic acetylcholine receptor; NRF2, nuclear factor erythroid 2-related factor 2; HO-1, heme oxygenase-1; Aβ, β-amyloid; SOD, superoxide dismutase; GPx, glutathione peroxidase; CAT, catalase; T-AOC, total antioxidant capacity; p.o., per os; GSH, glutathione; MDA, malondialdehyde; ERK, extracellular signal-regulated kinase; CHOP, C/EBP homologous proteins; APP/PS1, amyloid precursor protein/presenilin 1; PI3K, phosphoinositide-3 kinase; Akt, protein kinase B; GSK-3β, glycogen synthase kinase 3 beta; PARP-1, poly (ADP-ribose) polymerase 1; LPS, lipopolysaccharide; BAX, bcl-2 associated X protein; BACE1, beta-secretase 1; NF-kB, nuclear factor kappa B; ROS, reactive oxygen species; TLR4, toll-like receptor 4; TNF-a, tumor necrosis factor a; ARE, antioxidant response element; NQO1, NAD(P)H quinone oxidoreductase 1; Keap1, Kelch like ECH associated protein 1; IL-6, interleukin 6; IL-1β, interleukin 1β; H_2_O_2_, hydrogen peroxide; AChE, acetylcholinesterase; LPO, lipid peroxidation; L-Glu, L-glutamate; AlCl_3_, aluminum chloride; C. elegans, Caenorhabditis elegans; SKN-1, protein skinhead-1; FOXO, forkhead box protein O.

### 3.3 Chalcones

Chalcones are a class of flavonoid compounds consisting of two aromatic rings connected by a three-carbon α,β-unsaturated carbonyl system. They often contain hydroxyl (-OH) groups and other functional groups on the rings. The presence of the α,β-unsaturated carbonyl system imparts reactivity to chalcones, allowing them to undergo various chemical transformations (114). Chalcones have demonstrated a range of pharmacological properties, including antioxidant, anti-inflammatory, anticancer, antimicrobial, and neuroprotective activities. They interact with multiple cellular targets and signaling pathways, contributing to their diverse biological effects, while they activate NRF2 similarly to the previous categories.

2,3-dihydroxy-4,6-dimethoxychalcone (DDC) was shown to be protective against cellular oxidative damage and to reduce Aβ accumulation in primary mouse cortical neurons when administered at a dose of 30 µM in a 3-hour treatment. In a 24-hour treatment it appeared to increase HO-1 expression through activation of the NRF2/ARE pathway. However, neuronal death was not affected by DCC in either condition, suggesting that this mechanism is not the main neuroprotective mechanism of DCC (115).

Another compound of the chalcone class, hesperidin methylchalcone (HMC), was evaluated for its effect on the Neuro-2a cell model and in a Wistar rat model, where it showed a significant effect in reducing Aβ accumulation, protecting the cells from Aβ-induced toxicity. Also, HMC improved cognitive deficits in rats through enhancing cholinergic effects, inhibiting AChE, β-secretase, and caspase-3. Finally, HCM reduced Aβ-derived oxidative stress and reduced neuroinflammation by suppressing the NF-κB pathway and activating the NRF2/HO-1 pathway (116) (**Table 3**).

**Table 3:** Chalcone phytochemicals exerting neuroprotective activities in Alzheimer’s disease models.

Phytochemical tested	Plant	Concentration/Dose tested	Disease model	NRF2-related mechanism	Other observed AD-related effects	Reference
2,3-dihydroxy-4,6-dimethoxychalcone	*Perilla frutescens*	• 30 μM	• Primary cortical neurons	• NRF2/ARE activation	• Increase of HO-1 expression	(115)
Hesperidin methylchalcone	Citrus fruits	• 25-75 μg/ml • 25, 50 and 70 mg/kg	• Neuro-2a cells • Wistar rats	• Upregulation of NRF2/HO-1 pathway	• Downregulation of NF-kB • Inhibition of AChE/BuChE activity • Improvement of cognitive deficits caused by Aβ	(116)

NRF2, nuclear factor erythroid 2-related factor 2; ARE, antioxidant response element; HO-1, heme oxygenase-1; NF-kB, nuclear factor kappa B; AChE, acetylcholinesterase; BuChE, butyrylcholinesterase; Aβ, β-amyloid.

### 3.4 Terpenoids

Terpenoids are produced by plants, fungi, and some animals. Chemically, terpenoids are derived from a basic building block called isoprene. Isoprene units are combined to form different-sized carbon chains, which determine the classification of terpenoids. They are involved in plant defense mechanisms, serving as components of essential oils and contributing to the plant’s resistance against pathogens and herbivores. Terpenoids also have medicinal properties and are used in traditional medicine for their antimicrobial, anti-inflammatory, antioxidant, and anticancer effects (117). Similar to previous categories, terpenoids upregulate NRF2 leading to induction of phase II detoxifying enzymes and modulation of redox signaling.

Cycloastragenol, a triterpenoid saponin isolated from various legume species of the *Astragalus* genus, was administered orally at a dose of 20 mg/kg/day for 6 weeks to an AD mouse model induced by intracerebral injection of Aβ (5 μg/mouse). Its administration resulted in the enhancement of the expression of NRF2, HO-1, brain-derived neurotrophic factor (BDNF), phosphorylated tropomyosin-related kinase B receptor (p-TrkB) and neuronal nuclear protein (NeuN), while it reduced the expression of activated microglial cells and inflammatory cytokines. In addition, cycloastragenol improved apoptotic cell death and memory impairment (118).

Another terpenoid compound that showed therapeutic effect in AD is andrographolide (Andro), a diterpenoid isolated from the stem and leaves of *Andrographis paniculata*. Andro was tested in PC12 cells subjected to toxicity induced by aluminum maltolate [Al(mal)_3_] (700 µM) at doses of 1.25, 2.5, 5, 10, 20 and 40 µM and the results showed that the groups treated with 5 and 10 µM Andro had significantly increased cell viability from 67.4% to 91.9% and 91.2%, respectively, decreased expression of proteins related to neurotoxicity, such as APP and BACE1, while they had increased expression of NRF2, p62 and LC3 (microtubule-associated protein 1A/1B-light chain 3). Finally, an increase in the nuclear translocation of NRF2 and p62 and a decrease in the expression of Keap1 in the cytoplasm were observed (119).

Quinovic acid (QA), also a member of the terpene family, was studied for its activity against intracerebral Aβ_1-42_ injection-induced toxicity, cognitive deficits, and cholesterol dyshomeostasis in male C57BL/6J mice. The results showed that mice treated with Aβ_1-42_ had increased formation of Aβ oligomers, cholesterol accumulation in the brain and oxidative stress. However, QA administration significantly improved these outcomes by preventing oxidative stress through upregulating the NRF2/HO-1 pathway, reducing gliosis, neuroinflammatory mediators and apoptotic markers, and improving synaptic indices and spatial learning behaviors and memory (120).

Astaxanthin (ATX), mainly found in algae (*Haematococcus pluvialis*), yeasts (in yeast fungus *Xanthophyllomyces dendrorhous*) and animal species including trout and salmon, is a dark red xanthophylls carotenoid. It is also the most abundant flavonoid in propolis. In this study by Hafez et al. (121), it was investigated in AD-like rats induced by hydrated aluminum chloride (AlCl_3_^·^6H_2_O) solution in doses ranging from 5 to 15 mg/kg p.o. for 6 weeks. ATX was found to improve the MWM performance of rats, accompanied by suppressed accumulation of Aβ_1-42_ and MDA. It also inhibited AChE and monoamine oxidase, as well as BACE1. Finally, ATX also upregulated acetylcholine, serotonin, and NRF2, along with miRNA-124, proposing a good anti-AD activity (121). These neuroprotective effects were also corroborated in other studies which highlighted the implication of PI3K/Akt/GSK-3β/NRF2 signaling pathway in the mechanism of action of this phytochemical (122).

Furthermore, *in vivo* research in an experimental model of *C. elegans* showed that the compounds 2-butoxytetrahydrofuran (2-BTHF) and palmitic acid, diterpene glycosides derived from the sea cucumber *Holothuria scabra*, were able to extend the lifespan of *C. elegans* and enhance oxidative resistance, mainly through activation of DAF-16/FOXO/insulin/IGF (insulin-growth factor) and SKN-1/NRF2 signaling pathways (123) (**Table 4**). It is important to note that palmitic acid was administered at the rather low dose of 1 μg/mL, which might explain its beneficial effect, since this saturated fatty acid has been typically associated with negative effects on cell viability.

**Table 4:** Terpenoid phytochemicals exerting neuroprotective activities in Alzheimer’s disease models.

Phytochemical tested	Plant	Concentration/Dose tested	Disease model	NRF2-related mechanism	Other observed AD-related effects	Reference
Cycloastragenol	*Astragalus mongolica or Astragalus membranaceus*	• 20 mg/kg/day (p.o.)	• Aβ-induced AD mouse model	• Upregulation of NRF2	• Upregulation of HO-1, p-TrKB, BDNF, NeuN, p-JNK, p-P-38 and p-ERK	(118)
Androghrapholide	*Andrographis paniculata*	• 1.25, 2.5, 5, 10, 20 and 40 μM	• Al(Mal)_3_-induced PC12 cells	• Increase of NRF2 expression and nuclear translocation	• Increase of p62 levels and nuclear translocation	(119)
Quinovic acid	*Mitragyna rotundifolia*	• 50 mg/kg in a saline solution (i.p.) • 0.5, 10, 30, 55, 70, 85, 100 and 115 μM	• Aβ-induced AD mouse model • SH-SY5Y cells	• Upregulation of NRF2/HO-1 pathway	• Decrease of p53 expression and cholesterol accumulation • Downregulation of gliosis, IL-1, NF-kB, BAX, cleaved caspase-3 and cytochrome C • Improvement of spatial learning and memory behaviors	(120)
Astaxanthin	*Haematococcus pluvialis* *Xanthophyllomyces dendrorhous* Honeybee propolis Red-colored marine organisms	• 5, 10, 15 mg/kg in DMSO for 6 weeks (p.o.)	• AlCl_3_.6H_2_O solution-induced AD rat model	• Upregulation of NRF2	• Decreased Aβ_1-42_, BACE1, AChE, MAO, MDA • Increased miRNA-124, ACh, serotonin • Improved cognitive dysfunction	(121)
2-Butoxytetrahydrofuran and Palmitic acid	*Holothuria scabra*	• 1, 5 and 10 μg/ml	• *C. elegans*	• Upregulation of SKN-1/NRF2 pathway	• Upregulation of DAF-16/FOXO pathway	(123)

p.o., per os; Aβ, β-amyloid; AD, Alzheimer’s disease; NRF2, nuclear factor erythroid 2-related factor 2; HO-1, heme oxygenase-1; p-TrKB, phosphorylated tropomyosin receptor kinase B; BDNF, brain derived neurotrophic factor; NeuN, Fox-3, Rbfox3, or Hexaribonucleotide Binding Protein-3; p-JNK, phosphorylated C-Jun terminal kinase; p-ERK, phosphorylated extracellular signal-related kinase; Al(Mal)_3_, aluminum maltolate; i.p., intraperitoneally; IL-1, interleukin-1; NF-kB, nuclear factor kappa B; BAX, bcl-2 associated X protein; DMSO, dimethyl sulfoxide; AlCl_3_, aluminum chloride; BACE1, beta-secretase 1; AChE, acetylcholinesterase; MAO, monoamine oxidase; MDA, malondialdehyde; ACh, acetylcholine; C. elegans, Caenorhabditis elegans; SKN-1, protein skinhead-1; FOXO, forkhead box protein O.

### 3.5 Alkaloids

Alkaloids are another group of NRF2 activators. They are characterized by their nitrogen-containing heterocyclic structures and often exhibit potent physiological and pharmacological effects, as they can act as analgesics, stimulants, sedatives, anti-inflammatory agents, antimicrobials, anticancer agents, *etc.* (124).

A study investigating the beneficial effects of fangchinoline (FAN), a natural alkaloid, on HT22 cells with L-Glu-induced oxidative damage showed that FAN (1, 3, 5 μM) inhibited cell apoptosis in a dose-dependent manner and significantly decreased oxidative damage, reducing intracellular ROS levels and enhancing the activity of SOD and the endogenous antioxidant system. The underlying mechanism involved in these actions was the upregulation of NRF2/HO-1 levels through the downregulation of Keap1 protein expression (125).

Berberine, another natural alkaloid, was shown to decrease the activity of NF-κB and enhance the activity of the NRF2/HO-1 antioxidant system *in vitro* in HT22 cells exposed to Aβ_25-35_, providing neuroprotective effects. This mechanism was found in combination with other mechanisms, such as inhibition of cell apoptosis and reduction of oxidative stress, as well as improvement of mitochondrial membrane potential (126).

Finally, according to a study by Jiang et al. (127), i.p. administration of Rhynchophylline (Rhy) (10 or 20 mg/kg), an alkaloid found in the *Uncaria* species, to Aβ_1-42_-induced AD mice resulted in amelioration of cognitive impairment, limiting oxidative damage by reducing ROS, MDA and GSH levels and restoring the expression of NRF2 and the antioxidant enzymes it regulates in the frontal cortex and hippocampus of mice. Rhy was shown to exert its protective effects through activation of NRF2-ARE, as, when cells were transfected with NRF2 siRNA, Rhy activity was abolished (**Table 5**).

**Table 5:** Alkaloid phytochemicals exerting neuroprotective activities in Alzheimer’s disease models.

Phytochemical tested	Plant	Concentration/Dose tested	Disease model	NRF2-related mechanism	Other observed AD-related effects	Reference
Fangchinoline	*Stephania tetandra*	• 1, 3 and 5 μM	• Glutamate-induced HT22 cells	• Upregulation of NRF2/HO-1 • Downregulation of Keap1	• Increase of SOD activity	(125)
Berberine	*Berberis vulgaris, Berberis aristate*	• 0.5, 1 and 2 μM	• HT22 cells	• Sensitization and upregulation of NRF2	• Inhibition of apoptosis and intracellular ROS levels • Mitochondrial protection • Decrease of cytochrome C and cleaved caspase-3	(126)
Rhynchophylline	*Uncaria species*	• 10 or 20 mg/kg	• Aβ-induced AD mouse model • SH-SY5Y cells	• Upregulation of NRF2 and HO-1	• Decrease of MDA, ROS • Upregulation of NQO1 and GCLM	(127)

NRF2, nuclear factor erythroid 2-related factor 2; HO-1, heme oxygenase-1; Keap1, Kelch like ECH associated protein 1; SOD, superoxide dismutase; ROS, reactive oxygen species; Aβ, β-amyloid; AD, Alzheimer’s disease; MDA, malondialdehyde; NQO1, NAD(P)H quinone oxidoreductase 1; GCLM, glutamate-cysteine ligase regulatory subunit.

### 3.6 Polysaccharides

Polysaccharides are complex carbohydrates composed of long chains of the same or different monosaccharide units joined together by glycosidic bonds. They are abundant in nature and play crucial roles as structural components and energy storage molecules in organisms. In plants, they function as structural components in cell walls (*e.g.*, cellulose) or as storage molecules (*e.g.*, starch). In animals, polysaccharides such as glycogen serve as a storage form of glucose. Polysaccharides also play roles in cell recognition, immune response modulation, and as energy sources (128). The activation of NRF2 by polysaccharides has been reported in several studies, some of them described below.

Polysaccharides have shown therapeutic potential in reducing the pathogenesis of AD by demonstrating significant neuroprotective effects. According to a study by Murphy et al. (129), in which intranasal application of the polysaccharide Mini-GAGR, a 0.7-kDa cleavage product of low-acyl gellan gum, was administered, the levels and nuclear translocation of NRF2 were increased, through enhanced dissociation from its inhibitor, Keap1. An upregulation in NRF2-dependent antioxidant enzymes and growth associated protein 43 (GAP43) was also found. Administration was performed at a dose of 100 nmol/40μl Mini-GAGR (20μl/nostril) in 3xTg-AD mouse models (with an average weight of 25.5g) once daily for a period of 20 days. After 20 days of treatment, a reduction in p-tau and Aβ levels was observed in neurons *in vivo*, and memory improvement was also observed.

In a different study investigating the neuroprotective properties of *Amanita caesarea* polysaccharides (ACPS), it was found that pre-treatment with ACPS (2.5 or 5 μg/ml) in HT22 cells 3 hours before their exposure to L-Glu (25 mM), improved cell viability and oxidative stress by enhancing the nuclear levels of NRF2 and reducing the cytoplasmic levels of NRF2 and cytochrome C. In addition, it suppressed the expression of Keap1 protein and enhanced the expression of antioxidant enzymes such as HO-1, SOD and GCLC (130) (**Table 6**).

**Table 6:** Polysaccharide phytochemicals exerting neuroprotective activities in Alzheimer’s disease models.

Phytochemical tested	Plant	Concentration/Dose tested	Disease model	NRF2-related mechanism	Other observed AD-related effects	Reference
Mini-GAGR	*Gellan gum*	• 100 nmol/40 μl mini-GAGR (20 μl/nostril)	• 3xTg-AD mice	• Increase of NRF2 levels and activity • Dissociation of NRF2 from Keap1 • Phosphorylation and nuclear translocation of NRF2	• Decrease of ROS, p-tau and Aβ • Mitochondrial protection from oxidative damage • Memory improvement	(129)
Amanita caesarea polysaccharides	*Amanita caesarea*	• 2.5 or 5 μg/ml	• L-Glu exposed HT22 cells	• Upregulation of NRF2 nuclear levels • Reduction of cytoplasmic NRF2 levels • Keap1 suppression	• Upregulation of HO-1, SOD-1 and CLC • Downregulation of cytochrome C	(130)

AD, Alzheimer’s disease; NRF2, nuclear factor erythroid 2-related factor 2; Keap1, Kelch like ECH associated protein 1; ROS, reactive oxygen species; p-tau, phosphorylated protein tau; Aβ, β-amyloid; L-Glu, L-glutamate; HO-1, heme oxygenase-1; SOD-1, superoxide dismutase 1; CLC, cysteine ligase catalytic subunit.

### 3.7 Ginseng root compounds, ginsenosides

Ginsenosides are a group of bioactive compounds found in the roots of Panax ginseng and related plants. They are classified as triterpene saponins, consisting of a dammarane triterpene backbone with sugar moieties attached, and are known for their diverse pharmacological properties and health benefits. They have been studied for their anti-diabetic, neuroprotective, anti-inflammatory, antioxidant, anticancer, and immune-modulating properties, among others (131).

According to studies in AD mouse models, two steroidal glycosides of the Ginsenoside family, Ginsenoside K (CK) and Ginsenoside Rk3 (GRk3), showed promising neuroprotective and antioxidant effects. CK (20 and 40 mg/kg) was found to significantly improve memory and reduce cell apoptosis and oxidative stress in a mouse model of scopolamine hydrobromide-induced memory impairment. CK also showed the ability to inhibit the expression of Aβ, as well as the ability to enhance the activation of the NRF2/Keap1 pathway and the activity of the endogenous antioxidant system (132). GRk3, according to a study in a model of APP/PS1 mice, restored mitochondrial membrane potential, reduced intracellular ROS production and reduced neuronal apoptosis, through activation of the AMPK/NRF2 signaling pathway. It also improved learning and memory deficits *in vivo* (133). Additionally, another compound of the same family, Ginsenoside Re, was evaluated for its effect against Aβ-induced cytotoxicity and apoptosis in SH-SY5Y cells, and the results showed that at doses of 20, 25, and 30 μM, it was able to inhibit Aβ-mediated mitochondrial apoptosis, reduce cytochrome C release, and inactivate caspase-3/9, while it was also found to enhance NRF2 activation (134). Finally, gintonin, a glycolipoprotein also derived from ginseng root, was administered as a treatment to Aβ-induced mice at a dose of 100 mg/kg/day (p.o.) for four weeks. It resulted in the reduction of oxidative stress by enhancing the expression of NRF2 and HO-1, which led to a reduction in ROS levels and lipid peroxidation. Furthermore, it suppressed activated microglial cells and inflammatory mediators, thereby improving synaptic and memory functions in the brain of Aβ-compromised mice (135) (**Table 7**).

**Table 7:** Ginseng root phytochemicals exerting neuroprotective activities in Alzheimer’s disease models.

Phytochemical tested	Plant	Concentration/Dose tested	Disease model	NRF2-related mechanism	Other observed AD-related effects	Reference
Ginsenoside K	*Panax ginseng*	• 20 and 40 mg/kg	• Scopolamine hydrobromide-induced AD mice	• Activation of NRF2/Keap1 pathway	• Increase of SOD and GPx • Decrease of MDA, ROS and Aβ production	(132)
Ginsenoside Rk3	*Panax ginseng C.A. Meyer*	• 1 and 10 mg/kg	• APP/PS1 mouse model • PC12 cells	• AMPK/NRF2 activation	• Improvement of neuronal apoptosis and mitochondrial membrane potential *in vitro* • Decrease of ROS production *in vitro* • Increase of SOD and GSH *in vivo* • Improvement of memory deficit *in vivo* • Decrease of MDA production, apoptosis and glial cells activation *in vivo*	(133)
Ginsenoside Re	*Panax ginseng*	• 20, 25 and 30 μM	• SH-SY5Y cells	• NRF2 activation	• Elevation of Bcl-2/BAX ratio • Decrease of cytochrome C release and ROS production • Inactivation of caspase-3/9	(134)
Gintonin	*Panax ginseng*	• 100 mg/kg/day (p.o.)	• Aβ-induced AD mouse model • BV-2 cells	• Upregulation of NRF2/HO-1 expression	• Decrease of ROS and lipid peroxidation	(135)

AD, Alzheimer’s disease; NRF2, Nuclear factor erythroid 2-related factor 2; Keap1, Kelch like ECH associated protein 1; SOD, superoxide dismutase; GPx, glutathione peroxidase; MDA, malondialdehyde; ROS, reactive oxygen species; Aβ, β-amyloid; APP/PS1, amyloid precursor protein/presenilin 1; AMPK, AMP-activated protein kinase; GSH, glutathione; Bcl-2, B-cell lymphoma 2; BAX, bcl-2 associated X protein; p.o., per os; HO-1, heme oxygenase 1.

### 3.8 Other phytochemicals

*Bacopa monnieri* extract, which contains various compounds such as the alkaloids brahmin, nicotine, herpestine, bacosides A and B, saponins A, B and C, triterpenoid saponins, stigmastanol, β-stigasterol, α-alanine, aspartic acid, glutamic acid, and serine and pseudojumbogenin glycosides (136, 137), was tested at doses from 10 to 100 µg/ml and was shown to inhibit tau protein aggregation *in vitro* and acted as an antioxidant by restoring NRF2 levels in Neuro-2a cells. It also reduced p-tau load and GSK-3β phosphorylation in formaldehyde-stressed cells, suggesting that *Bacopa monnieri* could be considered a potent herb against tau protein phosphorylation and aggregation (138).

Another compound tested for its therapeutic activity against tau pathology in mouse fibroblasts is dimethyl fumarate (DMF), which is an inducer of the transcription factor NRF2. The results showed that DMF, at a dose of 100 mg/kg intragastrically (i.g.) daily for 3 weeks, induces NRF2 transcription through a mechanism involving Keap1 and PI3K/Akt/GSK-3β pathways. DMF modulates hippocampal GSK-3β activity, tau phosphorylation, neuronal injury, and inflammatory processes involved in astrogliosis, microgliosis, and proinflammatory cytokine production. These findings suggest that DMF may have neuroprotective effects beyond the Keap1/NRF2 axis, by inhibiting GSK-3β in a mouse model of tauopathy (75).

Melatonin, which, in addition to bacteria and eukaryotic cells, is also found in plant organisms (139), was able to inhibit p-tau aggregation-induced toxicity in Neuro-2a and N9 cells, as well as to reduce the levels of p-tau. It also enhanced the cellular levels of NRF2 and its nuclear translocation in response to oxidative stress. Melatonin also upregulated anti-inflammatory cytokines and activated MAP3K (mitogen activated protein kinase kinase kinase) in tau-compromised microglial cells and neurons, while downregulating the pro-inflammatory proteins IL-1β and COX-2 (140).

Oxyphylla A, a compound extracted from *Alpinia oxyphylla*, was tested in *in vivo* and *in vitro* AD models at various concentrations (12.5-100 µM) and the results showed that it could reduce the expression of APP and Aβ and alleviate cognitive deficits in SAMP8 mice. In addition, oxyphylla A exerted antioxidant activity through the Akt/GSK-3β and NRF2/Keap1/HO-1 pathways (141).

According to a study by Wang et al. (142), who investigated the therapeutic effects of the extract of *Ginkgo biloba 761* (EGb761) against Aβ-induced cell damage in an *in vitro* model of AD, it was found that EGb761 (25, 50, 100 and 250 μg/ml) resulted in ameliorating cell damage by inhibiting apoptosis, reducing ROS production and blood-brain barrier permeability, while enhancing the expression of Akt, NRF2 and HO-1.

Forsythoside A (FA) was also studied *in vitro* in an array of cell lines (Aβ_1-42_-treated N2a cells, erastin-treated HT22 cells and LPS-induced BV-2 cells), where it showed similar results. Specifically, it provoked decreased lipid peroxidation and iron deposition, and enhanced mitochondrial activity and dopaminergic signaling. Pro-inflammatory cytokines were found downregulated, including IL-6, NO and IL-1, contrasting the levels of anti-inflammatory cytokines, while NF-kB signaling was also diminished. The *in vivo* results in male APP/PS1 double transgenic AD mice proposed an amelioration of memory and cognitive deficits, accompanied by reduced Aβ deposition and p-tau levels in the brain. All these results significantly implicated the NRF2/GPx4 (glutathione peroxidase 4) axis (143).

*In vitro* studies in PC12 cells that had undergone oxidative damage and apoptosis induced by Aβ_25-35_ showed that the compounds (1E,4E)-1,5-bis(4-hydroxy-3-methoxyphenyl)penta-1,4-dien-3-one (CB) and (1E,4E)-1-(3,4-dimethoxyphenyl)-5-(4-hydroxy-3,5-dimethoxyphenyl)penta-1,4-dien-3-one (FE), two curcumin analogues, as well as sargahydroquinoic acid (SHQA), a marine natural compound found in various species of the brown macroalga genus *Sargassum*, showed significant neuroprotective effects by enhancing the expression and translocation of NRF2, as well as the expression of NRF2-regulated antioxidant enzymes (19, 144).

A study in AD rats administered with *Benincasa hispida* fruit extract, an extract rich in alkaloids, flavonoids, vitamins, volatile polysaccharides, hemicellulose polysaccharides, terpenes, sterols, amino acids and fatty acids (145), in doses of 250 and 500 mg/kg/day for a period of 16 days, showed, among other findings, that the antioxidant genes *Keap1/NRF2/HO-1* were upregulated (146).

3H-1,2-dithiole-3-thione (D3T) is a compound found naturally in cruciferous plants such as broccoli and cauliflower. When i.p. administered (10 and 20 mg/kg) in a Tg2576 mouse model of AD, it significantly improved cognitive deficit, reduced Aβ accumulation and oxidative damage, and upregulated NRF2 and HO-1 levels, promoting neurogenesis in the mouse hippocampus (147).

According to a study in N2a/APPswe cells (mouse Neuro 2a cells expressing human APP harboring the Swedish mutation), it was found that sulforaphane at concentrations of 1.25 and 2.5 µM increased the expression and promoted the nuclear translocation of NRF2 by reducing the DNA demethylation levels of the NRF2 promoter, while a significant reduction in the intracellular levels of Aβ_1-40_ and Aβ_1-42_ and an increase in SOD were also observed (148).

*In vivo* and *in vitro* research on the potential actions of osthole (OST), a coumarin derivative, against AD showed that OST had good neuroprotective effects, particularly through the NRF2 pathway. In L-Glu-induced HT22 cells, preincubation with 20 and 40 μM OST helped to enhance cell viability, reduce apoptosis, and inhibit caspase-3, caspase-8, and caspase-9. OST also reduced intracellular ROS levels, restored mitochondrial membrane potential, and upregulated the expression of NRF2 and its regulated proteins. In APP/PS1 transgenic mice, administration of 15 and 30 mg/kg OST for 8 weeks resulted in improved memory and cognitive behaviors, decreased Aβ and p-tau accumulation, and enhanced NRF2 and downstream proteins expression levels, including SOD-1 and HO-1 (149).

According to the results of the study by Nakhate et al. (150), i.p. administration of 0.5 and 1 mg/kg of the compound 5-hydroxy-2-methyl-1,4-naphthoquinone or plumbagin one hour before the first dose of STZ in a mouse model of STZ-induced AD, resulted in improving cognitive deficits, as assessed by MWM, through inhibition of the β-secretase enzyme and activation of the NRF2/ARE pathway.

Finally, treatment with *Hibiscus sabdariffa L.* extract (HSE), hydroxycitric acid (HCA) and isocitric acid (ICA) in a *C. elegans* model showed that HSE and HCA prolonged the lifespan of *C. elegans* by 24% and 6%, respectively, while ICA did not show similar efficacy. HSE and HCA also enhanced the nuclear localization of the transcription factors DAF-16/FOXO and SKN-1/NRF2 (151) (**Table 8**).

**Table 8:** Other phytochemicals exerting neuroprotective activities in Alzheimer’s disease models.

Phytochemical tested	Plant	Concentration/Dose tested	Disease model	NRF2-related mechanism	Other observed AD-related effects	Reference
*Bacopa monnieri* extract	*Bacopa monnieri*	• 10 to 100 μg/ml	• Neuro-2a tau-stressed cells	• Upregulation of NRF2	• Reduction of GSK-3β phosphorylation	(138)
Dimethyl fumarate	*Fumaria officinalis*	• 100 mg/kg (i.g.)	• MEFs from wild type or Keap1-deficient mice	• Disruption of Keap1/NRF2 axis by inhibition of GSK-3β	• Modulation of tau phosphorylation	(75)
Melatonin	*Scutellaria biacalensis, Tanacetum parthenium*, *Hypericum perforatum*, Common food	• 0.1 to 100 μM	• N9 microglia • Neuro-2a cells	• Cellular increase and nuclear translocation of NRF2	• Downregulation of GSK-3β	(140)
Oxyphylla A	*Alpinia oxyphylla*	• 12.5-100 μM	• N2a/APP cells • SAMP8 mice	• NRF2 activation through Akt-GSK-3β pathway	• Decrease of Keap1 • Decrease of APP and Aβ levels	(141)
EGb761	*Ginkgo biloba*	• 25, 50, 100 and 250 μg/ml	• SH-SY5Y cells	• Upregulation of NRF2	• Upregulation of HO-1, p-Akt and Bcl-2 • Decrease of caspase-3 and BAX	(142)
Forsythoside A	*Forsythia suspensa (Thunb.) Vahl.*	• 40 and 80 μM • 30 mg/kg	• Aβ_1-42_-treated N2a cells • Erastin-treated HT22 cells • LPS-induced BV-2 cells • APP/PS1 AD mouse model	• Activation of NRF2/GPx4 axis	• Decreased lipid peroxidation, iron deposition, IL-6, NO, IL-1, NF-kB signaling, ferroptosis, neuroinflammation • Increased mitochondrial activity, dopaminergic signaling, anti-inflammatory cytokines • Improvement of memory and cognitive deficits • Reduced Aβ deposition and p-tau in the brain	(143)
Curcumin analogues (FE, CB)	*Curcuma longa*	• 0.1, 1, 5, 10 and 20 μM	• Aβ_25-35_-treated PC12 cells	• Upregulation of NRF2/HO-1 • Decrease of Keap1	• Upregulation of SOD, CAT and Bcl-2 • Decrease of BAX and Cytochrome c	(144)
*Benincasa hispida* fruits extract	*Benincasa hispida*	• 250 and 500 mg/kg/day	• Young Male Sprague-Dawley AD rats	• Upregulation of *Keap1, NRF2, HO-1* genes	• Alleviation of ACh, dopamine and serotonin levels • Upregulation of antioxidant enzymes *i.e.* SOD, CAT, GSH • Decrease of MDA, TNF-a and IL-1β	(146)
3H-1,2-dithiole-3-thione	Cruciferous vegetables	• 10 and 20 mg/kg	• Tg2576 AD mouse model	• Upregulation of SIRT1/NRF2/HO-1	• Decrease of insoluble Aβ level	(147)
Sulphoraphane	Cruciferous vegetables	• 1.25 and 2.5 μM	• N2a/Wt cells • N2a/APPswe cells	• Upregulation of NRF2 • Nuclear translocation of NRF2 by decreasing DNA demethylation levels of its promoter	• Decrease of Aβ_1-40_, Aβ_1-42_ and intracellular Aβ_1-42_, ROS, MDA • Increase of SOD activity • Decrease of IL-1β, IL-6, NF-kB, p65, COX-2, iNOS	(148)
Osthole	Medicinal plants *(Cnidium monnieri, Angelica pubescens etc.)*	• 20 and 40 μM • 15 and 30 mg/kg	• L-Glu-induced HT22 cells • APP/PS1 transgenic mice	• Upregulation of NRF2 and its downstream proteins	• Upregulation of SOD and HO-1 • Inhibition of caspase-3, -8 and -9 • Reduction of ROS	(149)
Plumbagin	*Genus Plumbago*	• 0.5 and 1 mg/kg/day	• STZ-induced AD mice	• Upregulation of NRF2/ARE pathway	• Astrogliosis suppression • Inhibition of BACE1	(150)
*Hibiscus sabdariffa L*. extract, hydroxycitric acid	*Hibiscus sabdariffa*	• 0.25, 0.5 and 1 mg/ml	• *C. elegans*	• Increase of the nuclear localization of SKN-1/NRF2 and DAF-16/FOXO	• Life extension of *C. elegans*	(151)

NRF2, nuclear factor erythroid 2-related factor 2; GSK-3β, glycogen synthase kinase 3 beta; i.g., intragastrically; MEFs, mouse embryonic fibroblasts; Keap1, Kelch like ECH associated protein 1; APP, amyloid precursor protein; Akt, protein kinase B; Aβ, β-amyloid; HO-1, heme oxygenase-1; Bcl-2, B-cell lymphoma 2; BAX, bcl-2 associated X protein; LPS, lipopolysaccharide; APP/PS1, amyloid precursor protein/presenilin 1; AD, Alzheimer’s disease; GPx4, glutathione peroxidase 4; IL-6, interleukin-6; NO, nitric oxide; IL-1, interleukin-1; NF-kB, nuclear factor kappa B; p-tau, phosphorylated protein tau; SOD, superoxide dismutase; CAT, catalase; ACh, acetylcholine; GSH, glutathione; MDA, malondialdehyde; TNF-a, tumor necrosis factor a; SIRT1, sirtuin 1; ROS, reactive oxygen species; COX-2, cyclooxygenase 2; iNOS, inducible nitric oxide synthase; L-Glu, L-glutamate; STZ, streptozotocin; ARE, antioxidant response element; BACE1, beta-secretase 1; SKN-1, protein skinhead-1; FOXO, forkhead box protein O; C. elegans, Caenorhabditis elegans.

**Figure 4:**
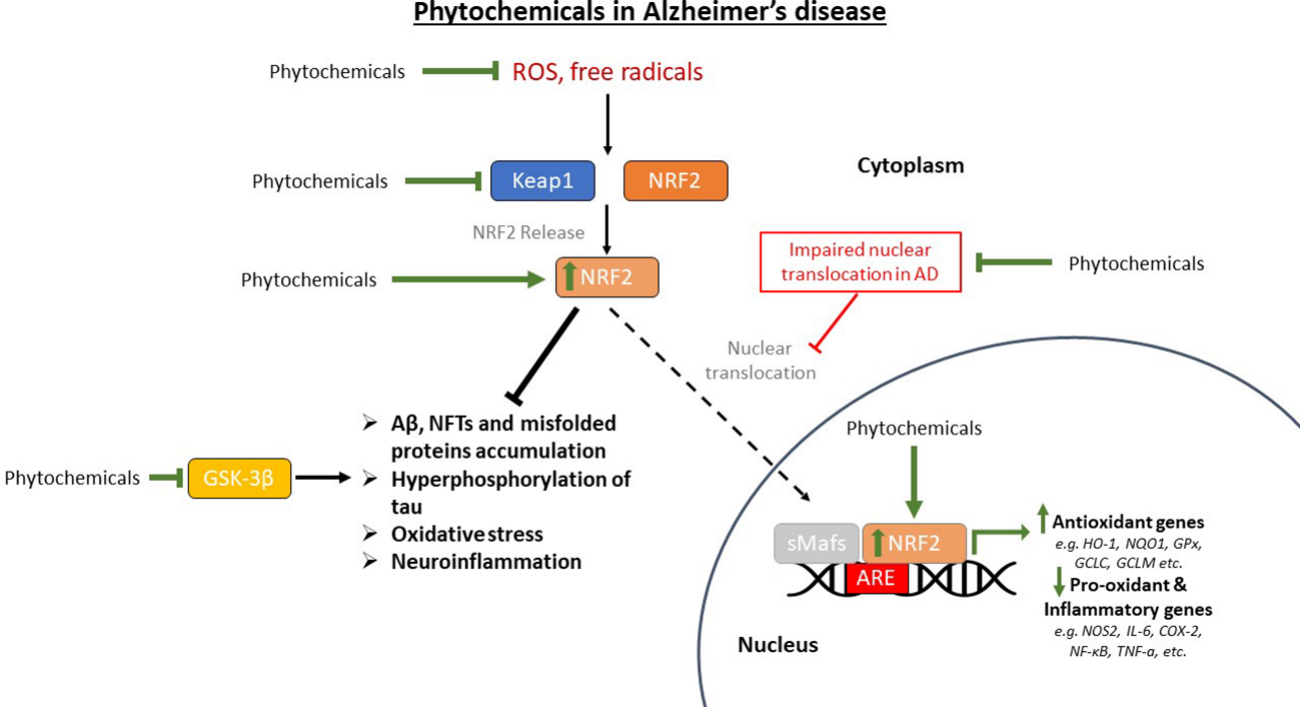
The restoration of the pathway of nuclear factor erythroid-derived 2-related factor 2 (NRF2) in Alzheimer’s disease (AD) models that are treated with phytochemicals. Phytochemicals scavenge reactive oxygen species (ROS) and free radicals reducing oxidative stress in the brain. They also upregulate NRF2, restoring its nuclear translocation that is inhibited in AD, thus upregulating antioxidant genes and downregulating pro-oxidant and pro-inflammatory genes. GSK-3β (glycogen synthase kinase-3β) has also been shown to be inhibited by certain phytochemicals, reducing the phosphorylation and subsequent aggregation of tau protein, along with the other AD pathological findings. *Aβ, amyloid beta; Keap1, Kelch-like ECH-associated protein 1; sMafs, small musculoaponeurotic fibrosarcoma proteins; ARE, antioxidant response element; HO-1, heme oxygenase-1; NQO1, NAD(P)H quinone oxidoreductase 1; GPx, glutathione peroxidase; GCLC, glutamate-cysteine ligase catalytic subunit; GCLM, glutamate-cysteine ligase regulatory subunit; NOS2, nitric oxide synthase 2; IL-6, interleukin-6; COX-2, cyclooxygenase 2; NF-κB, nuclear factor kappa beta; TNF-a, tumor necrosis factor-a; NFTs, neurofibrillary tangles*.

The main mechanisms of action of the previously discussed phytochemicals are summarized in **Figure 4**.

## 4 Discussion

AD is the most common cause of dementia and one of the main progressive neurodegenerative disorders worldwide. It is a multifactorial disease that occurs in middle and late age and affects parts of the brain that regulate thinking and memory, causing mild impairment at an early stage. As it progresses, AD leads to severe cognitive deficits and inability to respond to the environment or perform any activity of daily living. These disease features cause significant problems in the life of AD patients, as they become unable to manage finances, their household and any other task in their lives. Finding new treatment strategies for the unmet need that is AD is considered more essential than ever.

The pathophysiology of the disease is still partially understood, however, oxidative stress is viewed as one of the key regulators of its pathogenesis, as it contributes to aging and neurodegeneration, and thus, is considered a favorable therapeutic target for AD. One way to prevent oxidative damage is to upregulate the endogenous antioxidant system in neuronal cells. The NRF2/Keap1 signaling pathway is the main pathway of antioxidant defense and control of neuroinflammation, and its activation is considered very important for the treatment of the disease. Since NRF2-activating compounds have already been approved or are under approval for diseases such as multiple sclerosis (152–154), and Friedrich’s ataxia (155), phytochemicals that exert their actions through this transcription factor may comprise a novel therapeutic strategy against AD.

This review examined the effects of phytochemical antioxidants that act as NRF2 activators on various cell, mouse, rat, and *C. elegans* models of disease. The chemicals were administered at different dosages and by different routes of administration. Evidence for dose-dependent reductions in markers of oxidative stress and dose-dependent increases in viability were provided by several *in vitro* studies, conducted using HT22 (18, 103, 111, 125, 126, 130, 143, 149), SH-SY5Y (97, 109, 120, 127, 134, 142), PC12 (91, 101, 112, 119, 133, 144) and BV-2 cell lines (105, 108, 111, 143). These data indicate that phytochemicals exhibit general beneficial effects across multiple cell types, particularly in relation to their antioxidant and anti-inflammatory properties. It is important to note that the concentrations of phytochemicals used showed substantial variation between different studies, spanning from the low μM to the mM range. Although studies examining the effects of high phytochemical concentrations on cell viability were generally well-controlled (93, 95), it is worth considering that the practical application of such concentrations in humans may be restricted by solubility and bioavailability concerns. A second important aspect to consider for the administration of phytochemicals is that their beneficial effects require sufficient time to develop. This was particularly evident in *in vivo* rodent studies, in which phytochemicals were chronically administered (>30 days) to improve the behavioral and biochemical aspects of Aβ-induced toxicity. Similarly, the effects of Quercetin on PC12 cell viability were more pronounced as treatment time increased (101).

The substances examined proved effective in improving the pathobiological features of AD through different mechanisms related to the NRF2 factor as well as other activities. Upon oxidative damage, NRF2 is released from Keap1 and enters the nucleus where it dimerizes with small musculoaponeurotic fibrosarcoma proteins (sMafs) and binds to the ARE sequence of target genes. Of the phytochemicals discussed, HLF, plumbagin, and DDC significantly enhanced the expression of this pathway. Of these, plumbagin was administered to a STZ-challenged mouse model and was able to also reduce astrogliosis and block BACE1 expression. These results confirm that the NRF2/ARE signaling pathway is particularly important in oxidative damage and neuroinflammation.

The two key biological hallmarks of AD are the accumulation of Aβ plaques and the hyperphosphorylation of tau protein, which results in the aggregation of NFTs. Melatonin, oxyphylla A, CAPE, *Bacopa monnieri* extract, DMF and RosA induced NRF2 activation and reduced GSK-3β activity, whose overexpression is associated with both Aβ and tau related toxicity. These chemicals also enhanced GSK-3β phosphorylation at Ser9, which has been associated with the upregulation of antioxidant enzymes such as HO-1 and SOD. Of these compounds, CAPE and DMF were tested in *in vivo* models through different routes of administration, and with a significant difference in the tested doses, with CAPE doses being ten times smaller. RosA and oxyphylla A also enhanced the phosphorylation of Akt, which, according to studies precedes and regulates the phosphorylation of GSK-3β at Ser9 to keep it in an inactive state (156, 157). Folecitin, respectively, after two weeks of administration, significantly enhanced the phosphorylation of Akt, which also led to the stimulation of NRF2 and HO-1, but had no effect on the phosphorylation of GSK-3β.

In addition to Aβ and tau, neuroinflammation is an important factor in AD progression, and Aβ aggregation is capable of further stimulating the secretion of inflammatory cytokines. The compounds HMC, QA, umuhengerin, sulforaphane, isolicuritigenin, nobiletin and hesperitin activate NRF2 and downregulate the expression of NF-κB, which regulates the expression of IL-1β, IL-6 and TNF-a. The compounds were administered in different models and doses, but it is worth noting that nobiletin produced effects at a relatively low dose (10 mg/kg) orally in an AD model of Aβ-challenged mice. Additionally, nobiletin, hesperitin, umuhengerin and sulforaphane reduced ROS and MDA levels, while umuhengerin and QA also reduced cholesterol accumulation. A significant reduction in the expression of IL-1β and IL-6 was also observed by the polyphenolic extract of *Arabidopsis thaliana*, which also increased IL-4, IL-10 and IL-13, by sulforaphane and farrerol, resulting in reducing neuroinflammation. Of these phytochemicals, sulforaphane has also been tested in clinical trials that examined its efficacy in autistic-type behaviors (158, 159) and showed very good results in reducing and improving symptoms, through activation of the NRF2 pathway, combined with a favorable safety profile in children with autism. Also, sulforaphane is being currently tested in an ongoing clinical trial (National Library of Medicine [NLM], NTC04848792) aimed at enhancing the NRF2 pathway in the elderly by combining it with physical exercise. So far, results show that NRF2 activation and *NQO1*, *HO-1*, and *glutathione reductase* gene expression occur in response to acute exercise.

The remaining flavonoids have limited applicability due to their poor oral bioavailability, low water solubility and low membrane permeability, and therefore it is important to find a suitable way of administering these substances. In the research by Omidfar et al. (160), nanophytosomes were used to improve the solubility and bioavailability of hesperidin and hesperetin. By forming nanoparticles through the interaction of these compounds with Phospholipon 90G, their solubility was enhanced. Through oral administration, a marked increase in the maximum concentration of hesperidin and hesperetin after complexation with lipids, up to 4-fold, was observed. Therefore, the association of active substances and phospholipids can make an effective strategy to improve the therapeutic effect of these compounds.

Finally, ginsenosides along with curcumin analogs were also found to upregulate NRF2 and other antioxidant enzymes, including SOD and CAT, and increase the antiapoptotic protein Bcl-2, while also decreasing cytochrome C activity. Another important observation was that cycloastragenol and C3G increased ERK levels, which were decreased by Aβ toxicity. ERK belongs to the MAPKs, and has been shown to be important for the stress response and cell survival due to the anti-apoptotic signals it stimulates in neuronal cells (161).

## 5 Conclusions

Targeting the NRF2 signaling pathway by phytochemical antioxidant compounds that act as its activators shows great potential as a therapeutic strategy against AD. Polyphenols, flavonoids, chalcones, ginseng root compounds, terpenoids, alkaloids and other herbal compounds have a variety of beneficial effects and derive from plants and organisms that are an essential part of diet or that are very easily accessible, thus attracting interest as potential therapeutic agents. These compounds all demonstrated the ability to activate NRF2, while some also enhanced its nuclear translocation. In addition, the majority of compounds induced the expression of endogenous antioxidant enzymes such as SOD, CAT and GSH. NRF2 activation prevented or delayed disease pathogenesis and progression through regulation of multiple pathways involving ARE, ERK, HO-1, BACE1, phase II antioxidant enzymes, *etc.* in animal and cell models.

Currently, with the exception of sulforaphane, these antioxidants have not progressed to human clinical trials, however due to their reduced toxicity, clinical trial testing will likely be possible in the future. Therefore, further research is necessary to confirm their action and safety in humans. Finally, one of the disadvantages of these compounds, especially polyphenols and flavonoids, which also causes a limitation in their application, is that they show low bioavailability due to their poor water solubility, and for this reason, more research on how to deliver these compounds in new innovative ways, as tested using hesperetin and hesperidin nanophytosomes, is of utmost importance.

## Author contributions

GS conducted the literature search, identified relevant literature, organized the material and wrote the first draft of the manuscript. AM edited the manuscript and contributed in the writing of sections of the manuscript. MK conceived and designed the study, supervised the writing of the manuscript and edited the final manuscript. All authors contributed to the article and approved the submitted version.

## Glossary

**Table:**

2-BTHF	2-butoxytetrahydrofuran
a7nAcR	a7 nicotinic acetylcholine receptor
Aβ	beta amyloid
ABCA7	ATP-binding cassette sub-family A member 7
AChE	acetylcholinesterase
ACPS	Amanita caesarea polysaccharides
AD	Alhzeimer's disease
ADAM	a disintegrin and metalloproteinase
AGEs	advanced glycation end products
Akt	protein kinase B
AMPK	5'adenosine monophosphate-activated protein kinase
APLP1	amyloid precursor-like protein 1
APLP2	amyloid precursor-like protein 2
APOE	apolipoprotein E
APP/PS1	amyloid precursor protein/presenilin 1
APP	amyloid-beta precursor protein
ARE	antioxidant response element
ATX	astaxanthin
AβO	beta amyloid oligomers
BACE1	beta-secretase 1
BAX	bcl-2 associated X protein
BDNF	brain derived neurotrophic factor
BIN1	bridging Integrator 1
BuChE	butyrylcholinesterase
C. elegans	Caenorhabditis elegans
C3G	cyanidin-3-glucoside
CA	caffeic acid
CA	complanatoside A
CAPE	caffeic acid phenethyl ester
CAT	catalase
CB	(1E, 4E)-15-bis(4-hydroxy-3-methoxyphenyl)penta-1, 4-dien-3-one
CD33	sialic acid binding Ig-like lectin 3
CK	ginsenoside K
CLU	clusterin
CNS	central nervous system
COX-2	cyclooxygenase 2
CR1	complement receptor type 1
CTF-99	intracellular C-fragment 99
D3T	3H-1, 2-dithiole-3-thione
DDC	2,3-dihydroxy-4,6-dimethoxychalcone
DMF	dimethyl fumarate
DR6	death receptor 6
EGb761	extract of Ginkgo biloba 761
ERK	extracellular signal-regulated kinase
FA	Forsythoside A
FA-97	caffeic acid phenethyl ester 4-O-glucoside
FAN	fangchinoline
FE	(1E,4E)-1-(3,4-dimethoxyphenyl)-5-(4-hydroxy-3,5-dimethoxyphenyl)penta-1,4-dien-3-one
FOXO	forkhead box protein O
GAP43	growth associated protein 43
GCLC	glutamate-cysteine ligase catalytic subunit
GCLM	glutamate-cysteine ligase regulatory subunit
GPx	glutathione peroxidase
GPx4	glutathione peroxidase 4
GRk3	ginsenoside Rk3
GSH	glutathione
GSK3β	glycogen synthase kinase 3 beta
GWAS	genome-wide association studies
HCA	hydroxycitric acid
HLF	hawthorn leaf flavonoids
HMC	hesperidin methylchalcone
HO-1	heme oxygenase 1
HSE	Hibiscus sabdariffa L. extract
HSF-1	heat shock factor 1
i.c.v.	intracerebroventricular
i.g.	intragastrically
i.p.	intraperitoneally
IAB	isoastilbin
ICA	isocitric acid
IGF	insulin-growth factor
IL-1	interleukin-1
IL-10	interleukin-10
IL-13	interleukin-13
IL-1b	interleukin-1b
IL-4	interleukin-4
IL-6	interleukin-6
iNOS	inducible nitric oxide synthase
ISL	isoliquiritigenin
JNK	c-Jun N-terminal kinase
Keap1	Kelch like ECH associated protein 1
LC3	microtubule-associated protein 1A/1B-light chain 3
LDH	lactate dehydrogenase
L-Glu	L-glutamic acid
LPO	lipid peroxidation
LPS	lipopolysaccharide
MAP3K	mitogen activated protein kinase kinase kinase
MDA	malondialdehyde
MEFs	Mouse embryonic fibroblasts
MWM	Morris water maze
N2a/APPswe	mouse N2a cells transfected with the Swedish mutant form of APP
NeuN	Fox-3
Rbfox3	or Hexaribonucleotide Binding Protein-3
NF-kB	nuclear factor kappa B
NO	nitric oxide
NOS2	nitric oxide synthase 2
NQO1	NAD(P)H quinone oxidoreductase 1
Nrf2	nuclear factor erythroid 2-related factor 2
OS	oxidative stress
OST	osthole
PARP-1	poly (ADP-ribose) polymerase 1
p-ERK	phosphorylated extracellular signal-related kinase
PI3K	phosphoinositide 3-kinase
PICALM	phosphatidylinositol Binding Clathrin Assembly Protein
PINK1	phosphatase and tensin homologue-induced kinase 1
p-JNK	phosphorylated C-Jun terminal kinase
PSEN1	presenilin1
PSEN2	presenilin 2
p-tau	phosphorylated protein tau
p-TrkB	phosphorylated tropomyosin related kinase B
QA	quinovic acid
RAGE	receptor for advanced glycation end products
Rhy	Rhynchophylline
ROS	reactive oxygen species
RosA	rosmarinic acid
sAPPa	soluble secreted amyloid precursor protein alpha
sAPPβ	soluble secreted amyloid precursor protein beta
sAβ	soluble Aβ
SCOP	scopolamine
SHQA	sargahydroquinoic acid
Sita	sitagliptin
SKN-1	protein skinhead-1
SOD	superoxide dismutase
SOD1	superoxide dismutase 1
STZ	streprozotocin
T-AOC	total antioxidant capacity
TLR4	toll-like receptor 4
TNF-a	tumor necrosis factor a
TREM2	triggering receptor expressed on myeloid cells 2
TrKB	tropomyosin receptor kinase B
TYROB	tyrosine kinase-binding protein
